# Supplementary material for: A Survey of Chinese Pig Farms and Human Healthcare Isolates Reveals Separate Human and Animal Methicillin‐Resistant Staphylococcus aureus Populations
Source: Adv Sci (Weinh). 2021 Dec 11;9(4):2103388. doi: 10.1002/advs.202103388 (PMC8811834; doi:10.1002/advs.202103388)
Supplement: Supplementary file 1 — Supporting Information [file ADVS-9-2103388-s001.pdf]

ADVANCED SCIENCE
Open Access

Supporting Information

for *Adv. Sci.*, DOI: 10.1002/advs.202103388

A survey of Chinese pig farms and human healthcare isolates reveals separate human and animal MRSA populations

Geng Zou[1], Marta Matuszewska[2], Ming Jia[1], Jianwei Zhou[1], Xiaoliang Ba[2], Juan Duan[1], Caishi Zhang[3], Jian Zhao[1], Meng Tao[1], Jingyan Fan[1], Xiangming Zhang[4], Wenping Jin[4], Tianpen Cui[5], Xianyu Zeng[5], Min Jia[5], Xiaojuan Qian[6], Chao Huang[1], Wenxiao Zhuo[1], Zhiming Yao[1], Lijun Zhang[1], Shaowen Li[1], Lu Li[1,7,8], Qi Huang[1,7,8], Bin Wu[1,7,8], Huanchun Chen[1,7,8], Alexander W. Tucker[2], Andrew J. Grant[2], Mark A. Holmes[2] & Rui Zhou[1,7,8*]*

Supplementary Figures

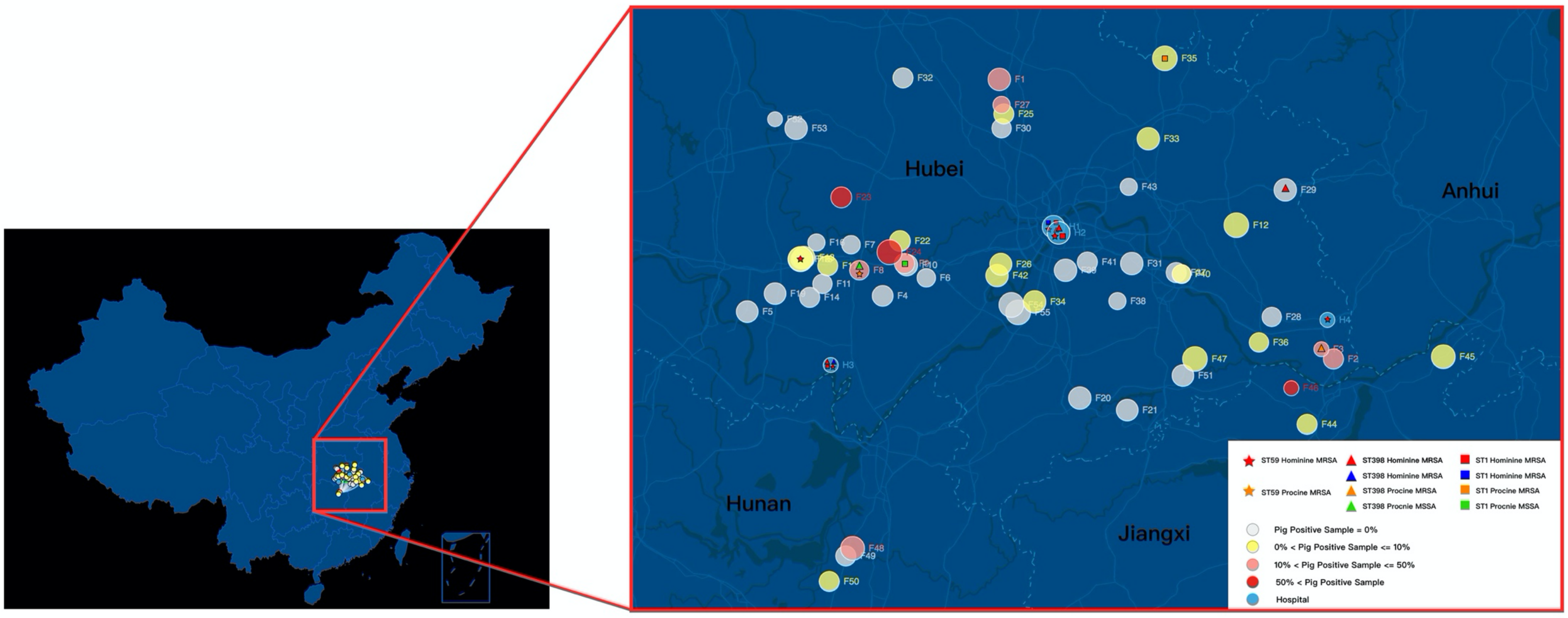

Figure S1. Distribution of the sampled pig farms and hospitals.

The distribution of the 55 sampled pig farms and 4 hospitals are illustrated on the map. The size of the circles reflects the size of the pig farm according to the number of sows.

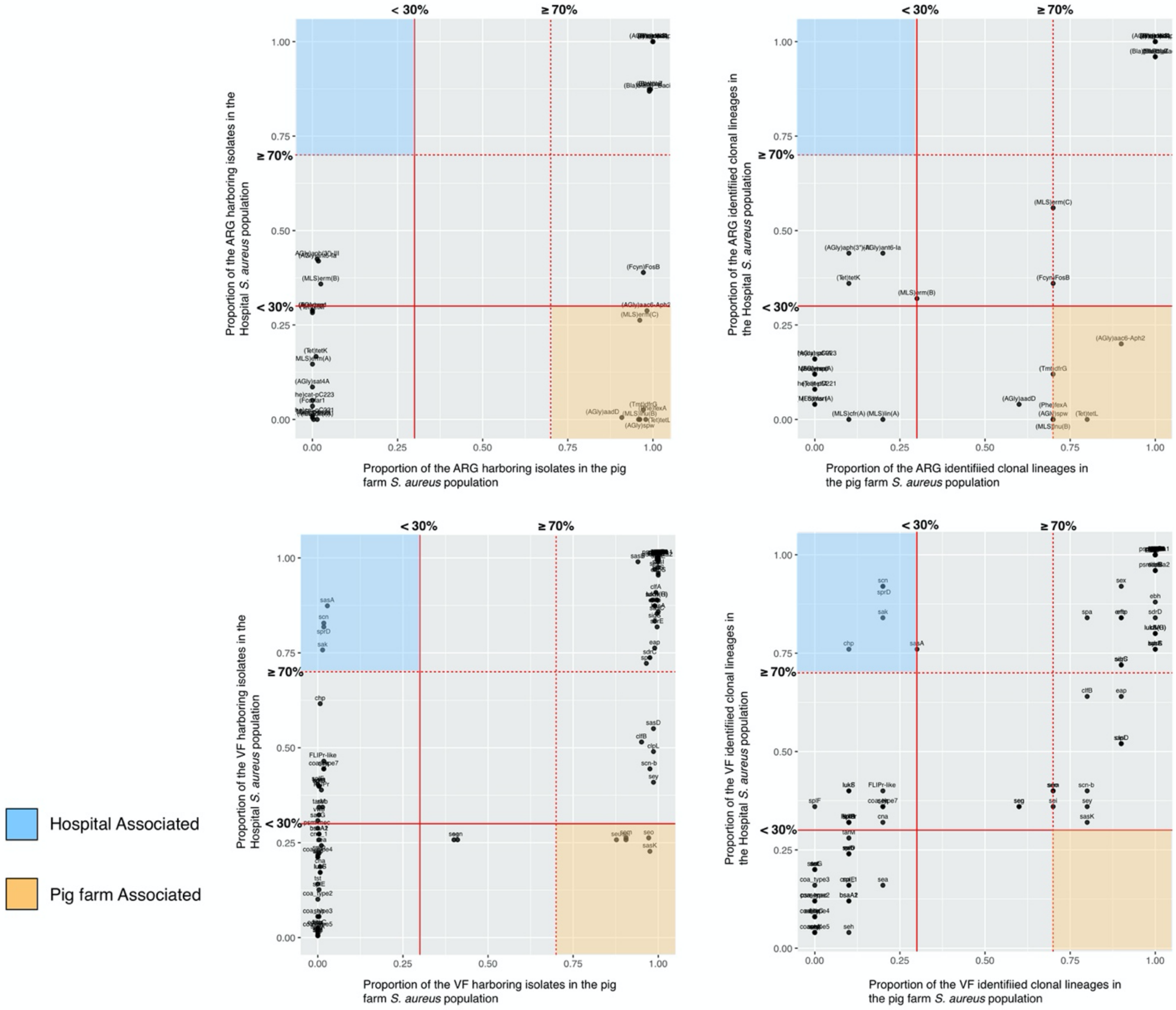

Figure S2. Determination of the pig farm associated ARGs and hospital associated VFs.

According to the distribution of the ARGs and VFs in different clonal lineages (STs) and the proportion of the isolates. The pig farm associated ARGs and hospital associated VFs were determined. The intersection of figures (A) and (B) were the selected ARGs, according to the results, the intersection of hospital associated was empty while that of the pig farm associated were *tetL*, *fexA*, *aac6-Aph2*, *spw*, *lnuB* and *dfrG*. The intersection of the figures (C) and (D) were the selected VFs, the intersection of pig farm was empty, and that of the hospital associated were *sprD*, *scn*, and *sak*.

(A)

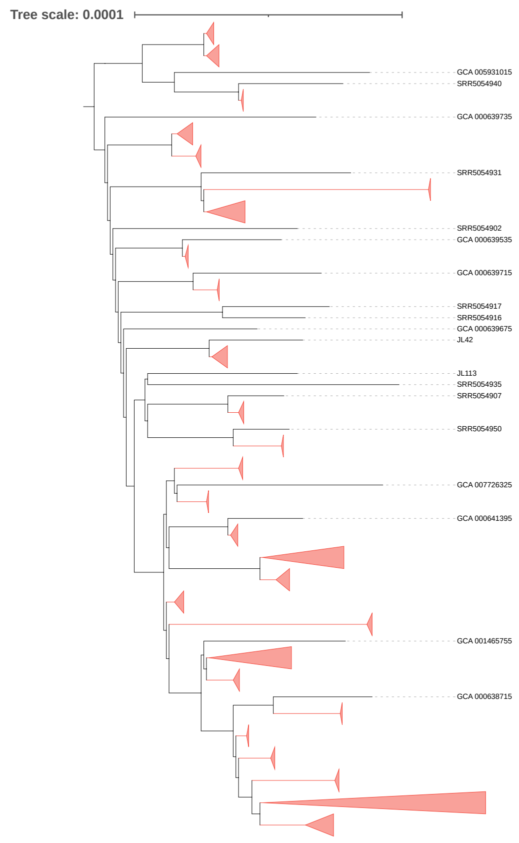

(B)

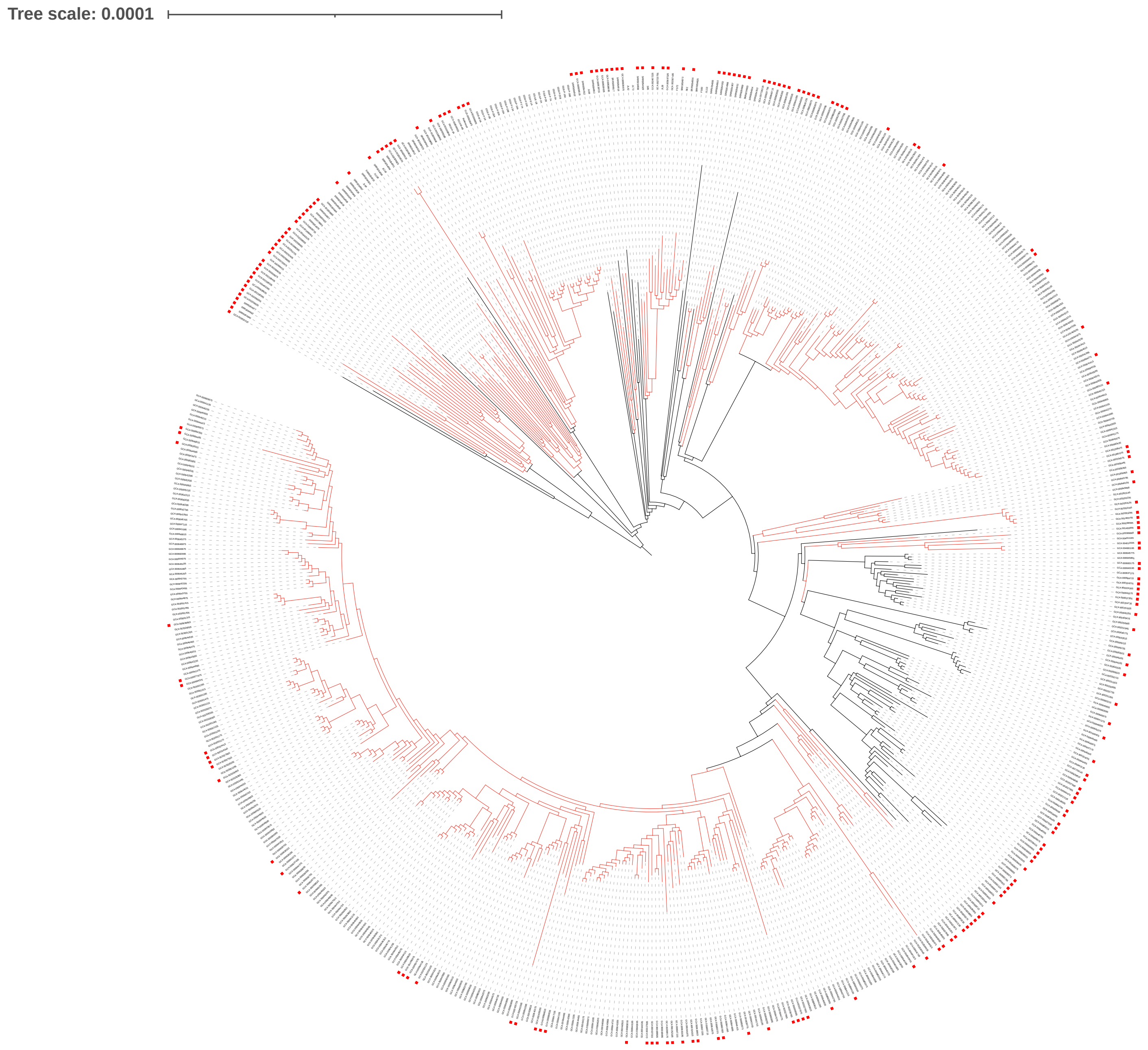

Figure S3 The RAxML tree of 582 high-assembled-quality ST398 isolates. (A) The branches with the length less than 0.00005 were collapsed (red color clades). (B) In each clade, if the collected date and host and location information was the same, only one of the isolates was selected (marked with red squares).

Table S1 The metadata of the *S. aureus* isolates in this study

ID	Source	Sample Source	Mec	ST	Spa	SCCmec
0116-H-1A	worker	F2	mecA	59	t437	IVa
0116-H-1B	worker	F2	mecA	59	t437	IVa
0116A-E-1A	Dust	F2	mecA	9	t899	ND
0116A-E-1B	Dust	F2	mecA	9	t899	ND
0116B-P-1A	Pig	F2	mecA	9	t899	ND
0116B-P-1B	Pig	F2	mecA	9	t899	ND
0116B-P-2A	Pig	F2	mecA	1376	t899	XII
0116B-P-2B	Pig	F2	mecA	9	t899	ND
0116B-P-3A	Pig	F2	mecA	9	t899	ND
0116B-P-3B	Pig	F2	mecA	9	t899	ND
0116B-P-4B	Pig	F2	mecA	9	t899	ND
0116B-P-5A	Pig	F2	mecA	9	t899	ND
0116B-P-5B	Pig	F2	mecA	9	t899	ND
0116B-P-6A	Pig	F2	mecA	9	t899	ND
0116B-P-6B	Pig	F2	mecA	9	t899	ND
0116B-P-7B	Pig	F2	mecA	1376	t899	XII
0116D-M-1B	Pig	F2	mecA	9	t899	XII
0116D-M-3A	Pig	F2	mecA	9	t899	XII
0116E-P-4A	Pig	F2	mecA	9	t899	XII
0116E-P-4B	Pig	F2	mecA	9	t899	XII
0116E-P-6A	Pig	F2	mecA	9	t899	XII
0116E-P-6B	Pig	F2	mecA	9	t899	XII
0116X-H-2A	worker	F2	mecA	9	t899	ND
0116X-H-2B	worker	F2	mecA	9	t899	ND
0213-M-1A	Pig	F3	mecA	9	t899	XII
0213-M-1C	Pig	F3	mecA	9	t899	XII
0213-M-4A	Pig	F3	mecA	398	t034	PF-SCC
0213-M-4B	Pig	F3	mecA	398	t034	PF-SCC
0213-M-5A	Pig	F3	mecA	9	t899	XII
0213-M-5B	Pig	F3	mecA	9	t899	XII
0213-P-10A	Pig	F3	mecA	9	t899	ND
0213-P-11A	Pig	F3	mecA	9	t899	ND
0213-P-12A	Pig	F3	mecA	9	t899	ND
0213-P-12B	Pig	F3	mecA	9	t899	ND
0213-P-12C	Pig	F3	mecA	9	t899	ND
0213-P-18A	Pig	F3	mecA	9	t899	ND
0213-P-18B	Pig	F3	ND	9	t899	MSSA
0213-P-1A	Pig	F3	mecA	9	t899	XII
0213-P-3B	Pig	F3	mecA	9	t899	ND
0213-P-3C	Pig	F3	mecA	9	t899	XII
0213-P-5B	Pig	F3	mecA	9	t899	ND
0213-P-7B	Pig	F3	mecA	9	t899	XII
0213-P-9A	Pig	F3	mecA	9	t899	XII
0213-P-9B	Pig	F3	mecA	9	t899	XII
0316-E-1A	Dust	F8	ND	398	t034	MSSA
0316-E-1B	Dust	F8	ND	398	t034	MSSA
0316-H-1B	worker	F8	mecA	9	t899	XII
0316-H-5A	worker	F8	ND	398	t034	MSSA
0316-H-6A	worker	F8	ND	1281	t14399	MSSA
0316-M-1A	Pig	F8	mecA	9	t899	XII
0316-M-1B	Pig	F8	mecA	9	t899	XII
0316-M-3A	Pig	F8	ND	398	t034	MSSA
0316-M-5A	Pig	F8	mecA	9	t899	XII
0316-M-5B	Pig	F8	mecA	9	t899	ND
0316-M-6A	Pig	F8	mecA	9	t899	XII
0316-M-6B	Pig	F8	mecA	9	t899	XII
0316-P-10A	Pig	F8	ND	398	t034	MSSA
0316-P-10B	Pig	F8	mecA	9	t899	XII
0316-P-10C	Pig	F8	ND	1281	t14399	MSSA
0316-P-11A	Pig	F8	ND	1281	t14399	MSSA
0316-P-13A	Pig	F8	ND	398	t034	MSSA
0316-P-15A	Pig	F8	mecA	9	t899	XII
0316-P-15B	Pig	F8	mecA	9	t899	XII
0316-P-16A	Pig	F8	ND	398	t034	MSSA
0316-P-16B	Pig	F8	ND	398	t034	MSSA
0316-P-17A	Pig	F8	ND	398	t034	MSSA
0316-P-18A	Pig	F8	ND	398	t034	MSSA
0316-P-19A	Pig	F8	ND	398	t034	MSSA
0316-P-19B	Pig	F8	ND	398	t034	MSSA
0316-P-21A	Pig	F8	mecA	9	t899	XII
0316-P-21B	Pig	F8	mecA	59	t441	V-VT
0316-P-23A	Pig	F8	mecA	9	t899	XII
0316-P-23B	Pig	F8	mecA	9	t899	XII
0316-P-25B	Pig	F8	ND	398	t034	MSSA

0316-P-27A	Pig	F8	mecA	9	t899	XII
0316-P-27B	Pig	F8	mecA	9	t899	XII
0316-P-28A	Pig	F8	ND	398	t034	MSSA
0316-P-2A	Pig	F8	ND	398	t034	MSSA
0316-P-2B	Pig	F8	ND	398	t034	MSSA
0316-P-30A	Pig	F8	ND	398	t034	MSSA
0316-P-3A	Pig	F8	ND	398	t034	MSSA
0316-P-3B	Pig	F8	ND	398	t034	MSSA
0316-P-4A	Pig	F8	ND	1281	t14399	MSSA
0316-P-7A	Pig	F8	ND	398	t034	MSSA
0316-P-8A	Pig	F8	mecA	9	t899	XII
0316-P-8B	Pig	F8	mecA	9	t899	XII
0318-E-2A	Dust	F9	mecA	9	t899	XII
0318-H-7A	worker	F9	mecA	9	t5390	XII
0318-H-7C	worker	F9	mecA	9	t5390	XII
0318-M-1A	Pig	F9	mecA	9	t4132	XII
0318-M-1B	Pig	F9	ND	1	t693	MSSA
0318-M-2B	Pig	F9	ND	1	t693	MSSA
0318-M-3A	Pig	F9	ND	1	t693	MSSA
0318-M-3B	Pig	F9	ND	1	t693	MSSA
0318-M-3C	Pig	F9	ND	1	t693	MSSA
0318-M-5A	Pig	F9	mecA	9	t899	ND
0318-M-6B	Pig	F9	mecA	9	t899	XII
0318-M-7B	Pig	F9	mecA	9	t899	XII
0318-P-10A	Pig	F9	mecA	9	t899	XII
0318-P-1A	Pig	F9	mecA	9	t18508	XII
0318-P-2C	Pig	F9	mecA	9	t899	XII
0318-P-3A	Pig	F9	mecA	9	t18508	XII
0318-P-5A	Pig	F9	mecA	9	t899	XII
0318-P-6A	Pig	F9	mecA	9	t18508	XII
0318-P-6B	Pig	F9	mecA	6553	t18508	XII
0323-P1-5A	Pig	F11	ND	1281	t14399	MSSA
0324-H-1A	worker	F12	mecA	9	t899	XII
0324-M-1A	Pig	F12	ND	5	t002	MSSA
0324-M-2A	Pig	F12	ND	5	t002	MSSA
0324-M-3A	Pig	F12	ND	5	t002	MSSA
0324-M-4A	Pig	F12	ND	5	t002	MSSA
0324-P-14A	Pig	F12	ND	9	t899	MSSA
0324-P-28A	Pig	F12	mecA	9	t899	XII
0324-P-9A	Pig	F12	mecA	9	t899	XII
0325-H-4A	worker	F13	mecA	9	t899	XII
0325-P-13A	Pig	F13	mecA	9	t899	XII
0406-EP-3A	Dust	F15	mecA	9	t899	XII
0406-H-14A	worker	F15	mecA	59	t437	IVa
0406-H-17A	worker	F15	mecA	9	t899	XII
0406-H-1A	worker	F15	mecA	9	t899	XII
0406-P-36A	Pig	F15	mecA	9	t899	XII
0406-P-39A	Pig	F15	mecA	9	t899	ND
0414-P-6A	Pig	F17	mecA	9	t899	XII
0422-H-3A	worker	F20	mecA	9	t899	XII
0425-H-4A	worker	F21	mecA	9	t899	XII
0428-H-7A	worker	F22	ND	9	t899	MSSA
0428-H-7B	worker	F22	ND	9	t899	MSSA
0428-P-26A	Pig	F22	mecA	9	t899	XII
0428-P-35A	Pig	F22	mecA	9	t899	XII
0428-P-40A	Pig	F22	mecA	9	t899	XII
0506-E-4B	Dust	F23	mecA	9	t899	XII
0506-E-4C	Dust	F23	mecA	9	t899	XII
0506-E-7A	Dust	F23	mecA	9	t899	XII
0506-H-1A	worker	F23	mecA	9	t899	XII
0506-H-1B	worker	F23	mecA	9	t899	XII
0506-H-1C	worker	F23	mecA	9	t899	XII
0506-H-7A	worker	F23	mecA	9	t899	XII
0506-M-1A	Pig	F23	mecA	9	t899	XII
0506-M-9B	Pig	F23	mecA	6556	t2445	XII
0506-P-10A	Pig	F23	mecA	9	t899	XII
0506-P-10B	Pig	F23	mecA	9	t899	XII
0506-P-10C	Pig	F23	mecA	9	t899	XII
0506-P-11A	Pig	F23	mecA	9	t899	ND
0506-P-12A	Pig	F23	mecA	9	t899	XII
0506-P-12B	Pig	F23	mecA	9	t899	XII
0506-P-14A	Pig	F23	mecA	9	t899	XII
0506-P-14B	Pig	F23	mecA	9	t899	XII
0506-P-15A	Pig	F23	ND	9	t899	MSSA
0506-P-16B	Pig	F23	mecA	9	t899	XII
0506-P-17A	Pig	F23	mecA	9	t899	XII
0506-P-17B	Pig	F23	ND	9	t899	MSSA

0506-P-19A	Pig	F23	mecA	9	t899	XII
0506-P-21B	Pig	F23	mecA	9	t899	XII
0506-P-21C	Pig	F23	mecA	9	t899	XII
0506-P-22B	Pig	F23	mecA	9	t899	ND
0506-P-23A	Pig	F23	mecA	9	t899	XII
0506-P-23B	Pig	F23	mecA	9	t899	XII
0506-P-25B	Pig	F23	mecA	9	t899	XII
0506-P-26A	Pig	F23	mecA	9	t899	XII
0506-P-26B	Pig	F23	ND	9	t899	MSSA
0506-P-27B	Pig	F23	ND	9	t899	MSSA
0506-P-27C	Pig	F23	mecA	9	t899	XII
0506-P-28A	Pig	F23	mecA	9	t899	XII
0506-P-2A	Pig	F23	mecA	9	t899	XII
0506-P-30C	Pig	F23	mecA	9	t899	XII
0506-P-32A	Pig	F23	mecA	9	t899	XII
0506-P-33B	Pig	F23	mecA	9	t899	XII
0506-P-34A	Pig	F23	mecA	9	t899	XII
0506-P-34B	Pig	F23	mecA	9	t899	XII
0506-P-36B	Pig	F23	ND	9	t899	MSSA
0506-P-38A	Pig	F23	mecA	9	t899	XII
0506-P-39B	Pig	F23	mecA	9	t899	XII
0506-P-3B	Pig	F23	mecA	9	t899	XII
0506-P-40A	Pig	F23	mecA	9	t899	XII
0506-P-40B	Pig	F23	mecA	9	t899	XII
0506-P-43B	Pig	F23	mecA	9	t899	XII
0506-P-44A	Pig	F23	mecA	9	t899	XII
0506-P-45A	Pig	F23	mecA	9	t899	XII
0506-P-49A	Pig	F23	mecA	9	t899	XII
0506-P-50A	Pig	F23	ND	9	t899	MSSA
0506-P-50B	Pig	F23	mecA	9	t899	XII
0506-P-51A	Pig	F23	mecA	9	t899	ND
0506-P-5B	Pig	F23	ND	9	t899	MSSA
0506-P-6A	Pig	F23	mecA	9	t899	XII
0506-P-6B	Pig	F23	mecA	9	t899	ND
0506-P-8A	Pig	F23	mecA	9	t899	XII
0506-P-8B	Pig	F23	mecA	9	t899	XII
0506-P-9A	Pig	F23	mecA	9	t899	XII
0509-H-1A	worker	F24	mecA	9	t899	XII
0509-H-1B	worker	F24	mecA	9	t899	XII
0509-H-5C	worker	F24	mecA	9	t899	XII
0509-H-6A	worker	F24	mecA	9	t899	XII
0509-H-6B	worker	F24	mecA	9	t899	XII
0509-H-6C	worker	F24	mecA	9	t899	XII
0509-M-11A	Pig	F24	mecA	9	t899	XII
0509-M-11B	Pig	F24	mecA	9	t899	XII
0509-M-12A	Pig	F24	mecA	9	t899	XII
0509-M-13A	Pig	F24	mecA	9	t899	XII
0509-M-14A	Pig	F24	mecA	9	t899	XII
0509-M-14B	Pig	F24	mecA	9	t899	XII
0509-M-17A	Pig	F24	ND	9	t899	MSSA
0509-M-17B	Pig	F24	mecA	9	t899	XII
0509-M-18A	Pig	F24	mecA	9	t899	XII
0509-M-18B	Pig	F24	mecA	9	t899	XII
0509-M-18C	Pig	F24	mecA	9	t899	XII
0509-M-19A	Pig	F24	mecA	9	t899	XII
0509-M-19B	Pig	F24	mecA	9	t899	XII
0509-M-19C	Pig	F24	mecA	9	t899	XII
0509-M-20A	Pig	F24	mecA	9	t899	XII
0509-M-20B	Pig	F24	mecA	9	t899	XII
0509-M-20C	Pig	F24	mecA	9	t899	XII
0509-M-21A	Pig	F24	mecA	9	t899	XII
0509-M-21B	Pig	F24	mecA	9	t899	XII
0509-M-21C	Pig	F24	mecA	9	t899	XII
0509-M-23A	Pig	F24	mecA	9	t899	XII
0509-M-23B	Pig	F24	mecA	9	t899	XII
0509-M-24A	Pig	F24	mecA	9	t899	XII
0509-M-24B	Pig	F24	mecA	9	t899	XII
0509-M-27A	Pig	F24	mecA	9	t899	XII
0509-M-27B	Pig	F24	mecA	9	t899	XII
0509-M-27C	Pig	F24	mecA	9	t899	XII
0509-M-28A	Pig	F24	mecA	9	t899	XII
0509-M-30A	Pig	F24	mecA	9	t899	XII
0509-M-30B	Pig	F24	ND	9	t899	MSSA
0509-M-4A	Pig	F24	ND	9	t899	MSSA
0509-M-6A	Pig	F24	mecA	9	t899	XII
0509-M-7A	Pig	F24	mecA	9	t899	XII
0509-M-9A	Pig	F24	mecA	9	t899	XII

0517-E-6A	Dust	F25	mecA	9	t899	XII
0517-H-2A	worker	F25	mecA	9	t899	XII
0517-P-13A	Pig	F25	mecA	9	t899	XII
0517-P-20A	Pig	F25	mecA	9	t899	XII
0517-P-49A	Pig	F25	mecA	9	t899	XII
0517-P-4A	Pig	F25	mecA	9	t899	XII
0520-P-14A	Pig	F26	mecA	9	t899	XII
0520-P-23A	Pig	F26	ND	9	t899	MSSA
0520-P-23B	Pig	F26	ND	9	t899	MSSA
0520-P-29A	Pig	F26	mecA	9	t899	XII
0520-P-29B	Pig	F26	mecA	9	t899	XII
0520-P-35A	Pig	F26	mecA	9	t899	XII
0520-P-9A	Pig	F26	mecA	9	t899	XII
0603-E-3A	Dust	F27	mecA	9	t899	XII
0603-H-1A	worker	F27	mecA	968	t899	ND
0603-H-2A	worker	F27	mecA	9	t899	XII
0603-H-5A	worker	F27	mecA	9	t899	XII
0603-P-13A	Pig	F27	mecA	9	t899	XII
0603-P-14A	Pig	F27	mecA	9	t899	XII
0603-P-16A	Pig	F27	mecA	9	t899	XII
0603-P-1A	Pig	F27	mecA	9	t899	XII
0603-P-21A	Pig	F27	mecA	9	t899	XII
0603-P-22A	Pig	F27	mecA	9	t899	XII
0603-P-2A	Pig	F27	mecA	9	t899	XII
0603-P-4A	Pig	F27	mecA	9	t899	XII
0603-P-7A	Pig	F27	mecA	9	t899	XII
0610-H-2A	worker	F29	mecA	398	t034	PF-SCC
0613-H-4A	worker	F31	mecA	9	t899	XII
0618-P-27A	Pig	F33	mecA	9	t899	XII
0619-P-1A	Pig	F34	mecA	9	t899	XII
0619-P-1B	Pig	F34	mecA	9	t899	XII
0622-M-7B	Pig	F35	mecA	1	t2207	IVi
0623-P-20A	Pig	F36	mecA	6555	t899	XII
0626-H-1A	worker	F37	mecA	9	t899	XII
0629-P-10A	Pig	F40	mecA	9	t899	XII
0629-P-10B	Pig	F40	mecA	9	t899	XII
0629-P-10C	Pig	F40	mecA	9	t899	XII
0629-P-11A	Pig	F40	mecA	9	t899	XII
0629-P-21A	Pig	F40	mecA	9	t899	XII
0629-P-21B	Pig	F40	mecA	9	t899	XII
0701-H-1A	worker	F41	mecA	9	t899	XII
0701-H-5A	worker	F41	mecA	6555	t899	XII
0701-H-5B	worker	F41	mecA	6555	t899	XII
0701-H-5C	worker	F41	mecA	6555	t899	XII
0702-P-16A	Pig	F42	mecA	9	t899	XII
1112-H-1A	worker	F44	mecA	9	t899	XII
1112-H-1B	worker	F44	mecA	9	t899	XII
1112-H-1C	worker	F44	mecA	9	t899	XII
1112-H-3B	worker	F44	mecA	9	t899	XII
1112-P1-1A	Pig	F44	mecA	9	t899	XII
1112-P1-3A	Pig	F44	mecA	9	t899	XII
1112-P1-9A	Pig	F44	mecA	9	t899	XII
1113-EP1-2B	Dust	F45	mecA	9	t899	XII
1113-EP1-2C	Dust	F45	mecA	9	t899	XII
1113-H-1B	worker	F45	mecA	9	t899	XII
1113-H-2A	worker	F45	mecA	9	t899	XII
1113-P1-10A	Pig	F45	mecA	9	t899	XII
1113-P1-11A	Pig	F45	mecA	6554	t899	XII
1113-P1-11B	Pig	F45	mecA	6554	t899	XII
1113-P1-8B	Pig	F45	mecA	9	t899	XII
1113-P1-9A	Pig	F45	mecA	9	t899	XII
1113-P3-11A	Pig	F45	mecA	9	t899	XII
1113-P3-5A	Pig	F45	mecA	9	t899	XII
1114-P-10A	Pig	F46	mecA	9	t899	XII
1114-P-10B	Pig	F46	mecA	9	t899	XII
1114-P-11B	Pig	F46	mecA	9	t899	XII
1114-P-12A	Pig	F46	mecA	9	t899	XII
1114-P-12B	Pig	F46	mecA	9	t899	XII
1114-P-12C	Pig	F46	mecA	9	t899	XII
1114-P-13A	Pig	F46	mecA	9	t899	XII
1114-P-14A	Pig	F46	mecA	9	t899	XII
1114-P-16B	Pig	F46	mecA	9	t899	XII
1114-P-17A	Pig	F46	mecA	9	t899	XII
1114-P-18A	Pig	F46	mecA	9	t899	XII
1114-P-18B	Pig	F46	mecA	9	t899	XII
1114-P-19A	Pig	F46	mecA	9	t1939	XII
1114-P-20A	Pig	F46	mecA	9	t1939	XII

1114-P-20B	Pig	F46	mecA	9	t899	XII
1114-P-20C	Pig	F46	mecA	9	t1939	XII
1114-P-21A	Pig	F46	mecA	9	t899	ND
1114-P-22B	Pig	F46	mecA	9	t899	XII
1114-P-3A	Pig	F46	mecA	9	t899	XII
1114-P-4A	Pig	F46	mecA	9	t899	XII
1114-P-4C	Pig	F46	mecA	9	t899	XII
1114-P-7A	Pig	F46	mecA	9	t1939	XII
1114-P-9C	Pig	F46	mecA	9	t1939	XII
1116-H-7A	worker	F47	mecA	9	t2922	XII
1116-P2-13A	Pig	F47	mecA	9	t899	XII
1123b-P1-10	Pig	F48	mecA	9	t899	XII
1123b-P1-15	Pig	F48	mecA	9	t899	XII
1123b-P1-16	Pig	F48	mecA	9	t899	XII
1123b-P1-17	Pig	F48	mecA	9	t899	XII
1123b-P1-4A	Pig	F48	mecA	9	t899	XII
1123b-P1-6A	Pig	F48	mecA	9	t899	XII
1124-H-1A	worker	F50	mecA	9	t899	XII
1124-P2-12A	Pig	F50	mecA	9	t899	XII
1213-M-4A	Pig	F1	mecA	9	t899	XII
1213-M-6A	Pig	F1	mecA	9	t899	XII
1213-P-37A	Pig	F1	mecA	9	t1939	XII
1213-P-46B	Pig	F1	mecA	9	t899	XII
1213-P-46C	Pig	F1	mecA	9	t899	XII
1213-P-48A	Pig	F1	mecA	9	t899	XII
1213-P-48B	Pig	F1	mecA	9	t899	XII
1213-P-50B	Pig	F1	mecA	9	t899	XII
1213-P-50C	Pig	F1	mecA	9	t1939	XII
1226a-H6-7A	worker	F55	mecA	9	t899	XII
1226a-H6-7B	worker	F55	mecA	9	t899	XII
FS1	Hospital	H1	mecA	59	t437	V-VT
FS100	Hospital	H1	mecA	59	t8347	IVa
FS101	Hospital	H1	mecA	22	t223	IVa
FS103	Hospital	H1	mecA	59	t437	IVa
FS106	Hospital	H1	mecA	59	t4350	IVa
FS108	Hospital	H1	mecA	239	t030	III
FS11	Hospital	H1	mecA	239	t030	III
FS12	Hospital	H1	mecA	239	t030	III
FS13	Hospital	H1	mecA	121	t8660	V
FS15	Hospital	H1	mecA	59	t437	IVa
FS16	Hospital	H1	mecA	239	t030	III
FS19	Hospital	H1	ND	188	t189	MSSA
FS2	Hospital	H1	mecA	59	t437	V-VT
FS20	Hospital	H1	ND	1	t127	MSSA
FS21	Hospital	H1	mecA	5	t5349	IVa
FS22	Hospital	H1	mecA	239	t030	III
FS23	Hospital	H1	mecA	22	t309	V-VT
FS24	Hospital	H1	mecA	59	t8347	IVa
FS25	Hospital	H1	mecA	239	t030	III
FS28	Hospital	H1	mecA	239	t037	III
FS3	Hospital	H1	mecA	239	t030	III
FS30	Hospital	H1	mecA	5	t5349	IVa
FS31	Hospital	H1	mecA	59	t437	IVa
FS32	Hospital	H1	mecA	59	t441	IVa
FS33	Hospital	H1	mecA	59	t437	IVa
FS34	Hospital	H1	mecA	59	t437	IVa
FS35	Hospital	H1	mecA	59	t437	IVa
FS36	Hospital	H1	mecA	630	t4549	H-SCC
FS37	Hospital	H1	mecA	239	t030	III
FS38	Hospital	H1	mecA	630	t4549	H-SCC
FS4	Hospital	H1	mecA	239	t030	III
FS40	Hospital	H1	mecA	4513	t437	IVa
FS42	Hospital	H1	mecA	59	t437	IVa
FS44	Hospital	H1	mecA	59	t437	V-VT
FS45	Hospital	H1	mecA	121	t8660	V
FS46	Hospital	H1	mecA	88	t10777	V-VT
FS47	Hospital	H1	mecA	59	t437	IVa
FS48	Hospital	H1	mecA	630	t4549	H-SCC
FS49	Hospital	H1	mecA	239	t030	III
FS5	Hospital	H1	mecA	121	t8660	V
FS51	Hospital	H1	mecA	59	t437	V-VT
FS52	Hospital	H1	mecA	59	t437	V-VT
FS53	Hospital	H1	mecA	630	t4549	H-SCC
FS54	Hospital	H1	mecA	338	t437	V-VT
FS55	Hospital	H1	mecA	6547	t437	IVa
FS56	Hospital	H1	mecA	1	t127	IVc
FS61	Hospital	H1	mecA	965	t062	IVc

FS62	Hospital	H1	mecA	8	t008	IVa
FS63	Hospital	H1	mecA	1	t114	IVg
FS64	Hospital	H1	mecA	121	t8660	V
FS68	Hospital	H1	mecA	59	t437	IVa
FS7	Hospital	H1	mecA	239	t030	III
FS71	Hospital	H1	mecA	338	t437	V-VT
FS72	Hospital	H1	mecA	398	t034	H-SCC
FS74	Hospital	H1	mecA	496	t002	IIa
FS75	Hospital	H1	mecA	239	t030	III
FS76	Hospital	H1	mecA	59	t437	IVa
FS78	Hospital	H1	ND	630	t652	MSSA
FS79	Hospital	H1	mecA	59	t437	IVa
FS83	Hospital	H1	mecA	398	t034	H-SCC
FS85	Hospital	H1	mecA	59	t437	IVa
FS87	Hospital	H1	mecA	239	t030	III
FS9	Hospital	H1	mecA	239	t030	III
FS91	Hospital	H1	mecA	509	t375	IVa
FS99	Hospital	H1	mecA	6548	t437	IVg
HMS1	Hospital	H4	ND	188	t189	MSSA
HMS10	Hospital	H4	mecA	5	t2460	IIa
HMS4	Hospital	H4	mecA	5	t2460	IIa
HMS5	Hospital	H4	mecA	5	t2460	IIa
HMS7	Hospital	H4	mecA	59	t437	IVa
JL10	Hospital	H3	ND	25	t078	MSSA
JL100	Hospital	H3	mecA	22	t309	V-VT
JL101	Hospital	H3	ND	2114	t701	MSSA
JL102	Hospital	H3	ND	630	t377	MSSA
JL103	Hospital	H3	mecA	59	t437	IVa
JL104	Hospital	H3	ND	188	t189	MSSA
JL105	Hospital	H3	ND	6544	t189	MSSA
JL106	Hospital	H3	ND	1	t177	MSSA
JL107	Hospital	H3	mecA	6546	t026	IVi
JL108	Hospital	H3	ND	398	t571	MSSA
JL109	Hospital	H3	mecA	59	t441	V-VT
JL11	Hospital	H3	ND	6	t701	MSSA
JL110	Hospital	H3	mecA	45	t116	IVa
JL111	Hospital	H3	ND	22	t309	MSSA
JL112	Hospital	H3	ND	188	t189	MSSA
JL113	Hospital	H3	ND	398	t011	MSSA
JL114	Hospital	H3	mecA	6549	t3385	IVa
JL115	Hospital	H3	ND	121	t2091	MSSA
JL116	Hospital	H3	ND	398	t571	MSSA
JL117	Hospital	H3	ND	944	t616	MSSA
JL118	Hospital	H3	mecA	1	t114	IVg
JL119	Hospital	H3	ND	188	t189	MSSA
JL12	Hospital	H3	mecA	1	t127	IVg
JL120	Hospital	H3	ND	944	t616	MSSA
JL121	Hospital	H3	ND	188	t189	MSSA
JL122	Hospital	H3	mecA	59	t437	IVa
JL123	Hospital	H3	ND	6	t701	MSSA
JL124	Hospital	H3	mecA	59	t437	IVa
JL125	Hospital	H3	mecA	59	t437	IVa
JL126	Hospital	H3	mecA	45	t116	IVa
JL127	Hospital	H3	ND	121	t1425	MSSA
JL128	Hospital	H3	mecA	45	t026	IVa
JL129	Hospital	H3	ND	25	t078	MSSA
JL13	Hospital	H3	ND	15	t084	MSSA
JL131	Hospital	H3	mecA	508	t050	IVi
JL132	Hospital	H3	ND	188	t2883	MSSA
JL133	Hospital	H3	mecA	45	t2519	IVa
JL135	Hospital	H3	mecA	59	t437	IVa
JL138	Hospital	H3	mecA	59	t437	IVa
JL139	Hospital	H3	mecA	59	t437	IVa
JL14	Hospital	H3	ND	7	t091	MSSA
JL141	Hospital	H3	mecA	59	t437	IVa
JL144	Hospital	H3	mecA	965	t062	IVc
JL147	Hospital	H3	mecA	59	t441	V-VT
JL149	Hospital	H3	mecA	965	t062	IVc
JL15	Hospital	H3	ND	22	t309	MSSA
JL154	Hospital	H3	mecA	45	t2519	IVa
JL16	Hospital	H3	mecA	59	t437	IVa
JL17	Hospital	H3	ND	7	t091	MSSA
JL18	Hospital	H3	ND	188	t189	MSSA
JL19	Hospital	H3	mecA	1	t127	IVg
JL2	Hospital	H3	mecA	1	t114	IVg
JL21	Hospital	H3	ND	188	t5229	MSSA
JL22	Hospital	H3	ND	188	t189	MSSA

JL23	Hospital	H3	ND	1281	07-06-17-21-	MSSA
JL24	Hospital	H3	mecA	59	t437	IVa
JL25	Hospital	H3	ND	5	t548	MSSA
JL26	Hospital	H3	mecA	508	t331	IVi
JL27	Hospital	H3	ND	188	t189	MSSA
JL28	Hospital	H3	mecA	398	t034	H-SCC
JL3	Hospital	H3	mecA	1	t114	IVg
JL30	Hospital	H3	mecA	59	t437	IVa
JL31	Hospital	H3	ND	25	t280	MSSA
JL32	Hospital	H3	mecA	59	t437	IVa
JL34	Hospital	H3	mecA	22	t309	V-VT
JL35	Hospital	H3	ND	1	t127	MSSA
JL37	Hospital	H3	ND	188	t189	MSSA
JL38	Hospital	H3	mecA	59	t441	V-VT
JL39	Hospital	H3	ND	1	t114	MSSA
JL4	Hospital	H3	mecA	5	t2460	IIa
JL40	Hospital	H3	mecA	6551	t1751	IVa
JL41	Hospital	H3	ND	5	t548	MSSA
JL42	Hospital	H3	ND	398	t034	MSSA
JL43	Hospital	H3	ND	188	t189	MSSA
JL44	Hospital	H3	mecA	45	t116	IVa
JL45	Hospital	H3	ND	121	t2092	MSSA
JL46	Hospital	H3	ND	508	t015	MSSA
JL47	Hospital	H3	mecA	45	t026	IVa
JL48	Hospital	H3	mecA	45	t015	IVa
JL49	Hospital	H3	mecA	59	t163	IVa
JL5	Hospital	H3	mecA	5	t2460	IIa
JL50	Hospital	H3	ND	25	t078	MSSA
JL51	Hospital	H3	ND	25	t3844	MSSA
JL52	Hospital	H3	ND	25	t081	MSSA
JL53	Hospital	H3	ND	188	t189	MSSA
JL54	Hospital	H3	ND	6	t701	MSSA
JL55	Hospital	H3	mecA	45	t026	IVa
JL56	Hospital	H3	mecA	45	t026	IVa
JL57	Hospital	H3	mecA	59	t437	IVa
JL58	Hospital	H3	mecA	22	t309	V-VT
JL59	Hospital	H3	ND	22	t309	MSSA
JL6	Hospital	H3	mecA	5	t2460	IIa
JL60	Hospital	H3	ND	121	t2019	MSSA
JL61	Hospital	H3	mecA	59	t437	IVa
JL62	Hospital	H3	ND	672	t3841	MSSA
JL63	Hospital	H3	ND	6545	08-16-02-25-	MSSA
JL64	Hospital	H3	mecA	59	t437	IVa
JL65	Hospital	H3	mecA	1	t127	IVg
JL66	Hospital	H3	ND	1	t127	MSSA
JL67	Hospital	H3	ND	623	t2182	MSSA
JL68	Hospital	H3	ND	188	t189	MSSA
JL69	Hospital	H3	ND	398	t034	MSSA
JL7	Hospital	H3	mecA	5	t2460	IIa
JL70	Hospital	H3	mecA	45	15-16-13-13-	IVa
JL71	Hospital	H3	mecA	45	t2519	IVa
JL72	Hospital	H3	mecA	59	t437	IVa
JL73	Hospital	H3	ND	398	t1451	MSSA
JL74	Hospital	H3	ND	188	t189	MSSA
JL75	Hospital	H3	mecA	45	t116	IVa
JL76	Hospital	H3	ND	6543	t2092	MSSA
JL77	Hospital	H3	ND	6	t701	MSSA
JL78	Hospital	H3	ND	25	t081	MSSA
JL79	Hospital	H3	mecA	59	t1950	IVa
JL8	Hospital	H3	mecA	5	t2460	IIa
JL80	Hospital	H3	mecA	59	t437	V-VT
JL81	Hospital	H3	mecA	338	t3590	V-VT
JL82	Hospital	H3	mecA	59	t437	IVa
JL83	Hospital	H3	ND	25	t081	MSSA
JL84	Hospital	H3	ND	398	t571	MSSA
JL85	Hospital	H3	mecA	45	t116	IVa
JL86	Hospital	H3	ND	5	t002	MSSA
JL87	Hospital	H3	mecA	22	t309	V-VT
JL88	Hospital	H3	mecA	45	t026	IVa
JL89	Hospital	H3	mecA	59	t437	V-VT
JL9	Hospital	H3	ND	5	t688	MSSA
JL90	Hospital	H3	ND	6552	t701	MSSA
JL91	Hospital	H3	mecA	59	t3736	IVa
JL92	Hospital	H3	mecA	1	t114	IVg
JL93	Hospital	H3	ND	6	t11363	MSSA
JL94	Hospital	H3	mecA	59	t437	IVa
JL95	Hospital	H3	mecA	59	t437	IVa

JL96	Hospital	H3	mecA	59	t437	IVa
JL97	Hospital	H3	ND	1	t127	MSSA
JL98	Hospital	H3	mecA	59	t437	IVa
JL99	Hospital	H3	mecA	59	t441	IVa
S1	Hospital	H2	mecA	239	t030	III
S10	Hospital	H2	mecA	5	26-17-34-34-	IIa
S12	Hospital	H2	mecA	239	t030	III
S16	Hospital	H2	mecA	30	t019	IVc
S19	Hospital	H2	mecA	239	t030	III
S2	Hospital	H2	mecA	630	t4549	H-SCC
S21	Hospital	H2	mecA	239	t030	III
S22	Hospital	H2	ND	2631	t3277	MSSA
S23	Hospital	H2	mecA	630	t4549	H-SCC
S24	Hospital	H2	mecA	59	t437	V-VT
S25	Hospital	H2	mecA	45	t116	IVa
S26	Hospital	H2	mecA	239	t030	III
S27	Hospital	H2	mecA	338	t437	V-VT
S30	Hospital	H2	mecA	239	t030	III
S31	Hospital	H2	mecA	59	t437	V-VT
S34	Hospital	H2	mecA	239	t030	III
S36	Hospital	H2	mecA	59	t437	V-VT
S37	Hospital	H2	mecA	59	t441	ND
S38	Hospital	H2	mecA	239	t037	III
S39	Hospital	H2	mecA	239	t2270	III
S40	Hospital	H2	mecA	239	t037	III
S41	Hospital	H2	mecA	5	t2460	IIa
S42	Hospital	H2	mecA	5	t2460	IIa
S43	Hospital	H2	mecA	45	t116	IVa
S45	Hospital	H2	mecA	239	t037	III
S46	Hospital	H2	mecA	239	t037	III
S47	Hospital	H2	mecA	239	t030	III
S48	Hospital	H2	mecA	239	t037	III
S5	Hospital	H2	mecA	239	t037	III
S51	Hospital	H2	mecA	59	t441	ND
S52	Hospital	H2	mecA	59	t441	ND
S53	Hospital	H2	mecA	239	t037	III
S54	Hospital	H2	mecA	239	t037	III
S57	Hospital	H2	mecA	59	t437	IVa
S58	Hospital	H2	mecA	5	t2460	IIa
S6	Hospital	H2	mecA	630	t4549	H-SCC
S60	Hospital	H2	mecA	5	t2460	IIa
S61	Hospital	H2	mecA	5	t2460	IIa
S62	Hospital	H2	mecA	630	t4549	H-SCC
S63	Hospital	H2	mecA	239	t037	III
S66	Hospital	H2	mecA	1	t114	IVg
S67	Hospital	H2	mecA	239	t037	III
S68	Hospital	H2	mecA	1821	t5554	V
S69	Hospital	H2	mecA	6550	t437	IVa
S7	Hospital	H2	mecA	59	t172	IVa
S70	Hospital	H2	mecA	59	t437	IVa
S71	Hospital	H2	mecA	239	t030	III
S72	Hospital	H2	mecA	239	t030	III
S73	Hospital	H2	mecA	239	t030	III
S74	Hospital	H2	mecA	59	t441	ND
S75	Hospital	H2	mecA	1	t114	IVg
S76	Hospital	H2	mecA	239	t030	III
S77	Hospital	H2	mecA	59	t437	IVa
S78	Hospital	H2	mecA	59	t437	IVa
S79	Hospital	H2	mecA	239	t030	III
S8	Hospital	H2	mecA	59	t441	ND
S80	Hospital	H2	mecA	338	t437	V-VT
S81	Hospital	H2	mecA	239	t030	III
S82	Hospital	H2	mecA	398	t034	H-SCC
S85	Hospital	H2	mecA	398	t034	H-SCC
S87	Hospital	H2	mecA	239	t030	III
S9	Hospital	H2	mecA	59	t172	IVa
S91	Hospital	H2	mecA	239	t037	III

ND: not detected

Table S2 The metadata of the international ST398 collection used for phylogenetic analysis

ID/NCBI Assembly_ID/SRA_access_ No.	assigned phylogenetic lineage	location	host	mecA	Date
0213-M-4A	10	China	Porcine	MRSA	2017
0213-M-4B	10	China	Porcine	MRSA	2017
0316-E-1A	10	China	Environmental	MSSA	2017
0316-E-1B	10	China	Environmental	MSSA	2017
0316-H-5A	10	China	Hominine	MSSA	2017
0316-M-3A	10	China	Porcine	MSSA	2017
0316-P-10A	10	China	Porcine	MSSA	2017
0316-P-13A	10	China	Porcine	MSSA	2017
0316-P-16A	10	China	Porcine	MSSA	2017
0316-P-16B	10	China	Porcine	MSSA	2017
0316-P-17A	10	China	Porcine	MSSA	2017
0316-P-18A	10	China	Porcine	MSSA	2017
0316-P-19A	10	China	Porcine	MSSA	2017
0316-P-19B	10	China	Porcine	MSSA	2017
0316-P-25B	10	China	Porcine	MSSA	2017
0316-P-28A	10	China	Porcine	MSSA	2017
0316-P-2A	10	China	Porcine	MSSA	2017
0316-P-2B	10	China	Porcine	MSSA	2017
0316-P-30A	10	China	Porcine	MSSA	2017
0316-P-3A	10	China	Porcine	MSSA	2017
0316-P-3B	10	China	Porcine	MSSA	2017
0316-P-7A	10	China	Porcine	MSSA	2017
0610-H-2A	10	China	Hominine	MRSA	2017
FS72	21	China	Hominine	MRSA	2018
FS83	21	China	Hominine	MRSA	2018
GCA_000009585.1	48	Netherlands	Hominine	MRSA	2006
GCA_000252405.2	5	Dominica	Hominine	MSSA	2007
GCA_000258685.1	5	USA	Hominine	MSSA	2004
GCA_000296595.1	47	Canada	Hominine	MRSA	2008
GCA_000401595.1	48	Switzerland	Bovine	MRSA	2008
GCA_000443125.1	5	France	Hominine	MSSA	2010
GCA_000443245.1	47	Netherlands	Hominine	MRSA	2010
GCA_000443265.1	49	Netherlands	Porcine	MSSA	2010
GCA_000443285.1	5	France	Hominine	MSSA	2009
GCA_000455725.2	48	France	Hominine	MRSA	2008
GCA_000577675.1	48	Netherlands	Porcine	MRSA	2005
GCA_000636255.1	48	Belgium	Murine	MRSA	2008
GCA_000636275.1	48	Netherlands	Murine	MSSA	2008
GCA_000636295.1	48	Netherlands	Murine	MRSA	2008
GCA_000636315.1	48	Netherlands	Murine	MRSA	2008
GCA_000636375.1	47	Netherlands	Hominine	MRSA	2008
GCA_000636415.1	47	Netherlands	Hominine	MRSA	2006
GCA_000636435.1	47	Netherlands	Hominine	MSSA	2007
GCA_000636455.1	44	Netherlands	Hominine	MRSA	2007
GCA_000636495.1	47	Netherlands	Hominine	MSSA	2008
GCA_000636535.1	47	Netherlands	Hominine	MRSA	2008
GCA_000636655.1	47	Netherlands	Hominine	MRSA	2011
GCA_000636675.1	47	Netherlands	Hominine	MRSA	2012
GCA_000636755.1	44	Netherlands	Porcine	MRSA	2007
GCA_000636775.1	44	Netherlands	Porcine	MRSA	2007
GCA_000637255.1	48	Germany	Galline	MRSA	2009
GCA_000637275.1	47	Germany	Galline	MRSA	2010
GCA_000637315.1	48	Germany	Galline	MRSA	2010
GCA_000637355.1	47	Germany	Galline	MRSA	2010
GCA_000637435.1	48	Netherlands	Bovine	MRSA	2011
GCA_000637475.1	48	Germany	Galline	MRSA	2011
GCA_000637635.1	47	Netherlands	Porcine	MRSA	2007
GCA_000637795.1	55	Netherlands	Porcine	MSSA	2007
GCA_000637855.1	44	Netherlands	Porcine	MSSA	2007
GCA_000637875.1	49	Netherlands	Porcine	MSSA	2007
GCA_000637895.1	49	Netherlands	Porcine	MSSA	2007
GCA_000637935.1	44	Netherlands	Porcine	MRSA	2007
GCA_000637955.1	44	Netherlands	Porcine	MSSA	2007
GCA_000638475.1	10	China	Galline	MSSA	2011
GCA_000638535.1	48	Spain	Hominine	MRSA	2009
GCA_000638635.1	5	Spain	Hominine	MSSA	2009
GCA_000638695.1	48	Spain	Hominine	MRSA	2009

GCA_000638715.1	48	Spain	Hominine	MRSA	2009
GCA_000638755.1	48	Spain	Hominine	MRSA	2009
GCA_000638775.1	48	Spain	Equine	MRSA	2005
GCA_000638815.1	48	Spain	Hominine	MRSA	2009
GCA_000638835.1	47	Spain	Hominine	MRSA	2009
GCA_000638855.1	48	Spain	Equine	MRSA	2011
GCA_000638895.1	48	Spain	Hominine	MRSA	2011
GCA_000638915.1	48	Austria	Galline	MRSA	2009
GCA_000638935.1	48	Germany	Galline	MRSA	2009
GCA_000639015.1	47	Netherlands	Bovine	MRSA	2007
GCA_000639035.1	47	Netherlands	Bovine	MRSA	2008
GCA_000639055.1	47	Netherlands	Bovine	MRSA	2008
GCA_000639075.1	42	Netherlands	Porcine	MRSA	2010
GCA_000639095.1	44	Netherlands	Porcine	MRSA	2010
GCA_000639115.1	44	Netherlands	Hominine	MRSA	2010
GCA_000639255.1	48	Netherlands	Hominine	MRSA	2010
GCA_000639315.1	48	Netherlands	Bovine	MRSA	2010
GCA_000639375.1	47	Netherlands	Galline	MRSA	2007
GCA_000639415.1	47	Netherlands	Meleagrine	MRSA	2008
GCA_000639435.1	47	Netherlands	Porcine	MRSA	2008
GCA_000639515.1	10	China	Feline	MSSA	2009
GCA_000639535.1	28	China	Feline	MSSA	2011
GCA_000639555.1	10	China	Porcine	MSSA	2010
GCA_000639575.1	5	Trinidad and Tobago	Hominine	MSSA	2013
GCA_000639635.1	32	China	Rodentia	MSSA	2010
GCA_000639675.1	33	China	Rodentia	MSSA	2010
GCA_000639695.1	32	China	Rodentia	MSSA	2011
GCA_000639715.1	31	China	Rodentia	MSSA	2011
GCA_000639735.1	13	China	Rodentia	MSSA	2011
GCA_000639755.1	48	Germany	Bovine	MRSA	2008
GCA_000639775.1	48	Germany	Bovine	MRSA	2008
GCA_000639815.1	47	Germany	Bovine	MRSA	2011
GCA_000639895.1	48	Belgium	Galline	MRSA	2006
GCA_000639935.1	48	Belgium	Galline	MRSA	2006
GCA_000639955.1	47	Spain	Hominine	MRSA	2010
GCA_000640175.1	48	Germany	Meleagrine	MRSA	2009
GCA_000640195.1	47	Austria	Meleagrine	MRSA	2009
GCA_000640215.1	47	Germany	Meleagrine	MRSA	2009
GCA_000640235.1	42	Germany	Meleagrine	MRSA	2009
GCA_000640255.1	47	Italy	Meleagrine	MRSA	2009
GCA_000640275.1	47	Germany	Meleagrine	MRSA	2009
GCA_000640475.1	47	Spain	Porcine	MRSA	2007
GCA_000640555.1	44	Netherlands	Hominine	MRSA	2008
GCA_000640575.1	48	Netherlands	Hominine	MRSA	2008
GCA_000640595.1	48	Netherlands	Hominine	MRSA	2008
GCA_000640615.1	44	Netherlands	Hominine	MRSA	2008
GCA_000640655.1	48	Netherlands	Hominine	MRSA	2008
GCA_000640675.1	48	Netherlands	Hominine	MRSA	2008
GCA_000640695.1	48	Netherlands	Hominine	MRSA	2008
GCA_000640735.1	44	Netherlands	Hominine	MRSA	2008
GCA_000640755.1	48	Netherlands	Hominine	MRSA	2008
GCA_000640775.1	48	Netherlands	Hominine	MRSA	2008
GCA_000640815.1	48	Netherlands	Hominine	MRSA	2008
GCA_000640835.1	44	Netherlands	Hominine	MRSA	2008
GCA_000640855.1	48	Netherlands	Hominine	MRSA	2008
GCA_000640895.1	44	Netherlands	Hominine	MRSA	2008
GCA_000640915.1	44	Netherlands	Hominine	MRSA	2008
GCA_000640955.1	44	Netherlands	Hominine	MRSA	2008
GCA_000640975.1	48	Netherlands	Hominine	MRSA	2008
GCA_000640995.1	44	Netherlands	Hominine	MRSA	2008
GCA_000641015.1	44	Netherlands	Hominine	MRSA	2008
GCA_000641035.1	48	Netherlands	Hominine	MRSA	2008
GCA_000641055.1	44	Netherlands	Hominine	MRSA	2008
GCA_000641095.1	48	Netherlands	Hominine	MRSA	2008
GCA_000641115.1	48	Netherlands	Hominine	MRSA	2008
GCA_000641135.1	48	Netherlands	Hominine	MRSA	2008
GCA_000641155.1	44	Netherlands	Hominine	MRSA	2008
GCA_000641175.1	44	Netherlands	Hominine	MRSA	2008
GCA_000641215.1	48	Netherlands	Hominine	MRSA	2008
GCA_000641235.1	44	Netherlands	Hominine	MRSA	2008
GCA_000641255.1	48	Netherlands	Hominine	MRSA	2009
GCA_000641275.1	48	Netherlands	Hominine	MRSA	2009

GCA_000641295.1	48	Netherlands	Hominine	MRSA	2009
GCA_000641315.1	48	Netherlands	Hominine	MRSA	2009
GCA_000641335.1	48	Netherlands	Hominine	MRSA	2009
GCA_000641355.1	48	Netherlands	Hominine	MRSA	2008
GCA_000641395.1	41	Netherlands	Hominine	MRSA	2008
GCA_000641415.1	44	Netherlands	Hominine	MRSA	2008
GCA_000641435.1	44	Netherlands	Hominine	MRSA	2008
GCA_000641475.1	48	Netherlands	Hominine	MRSA	2008
GCA_000641495.1	48	Netherlands	Hominine	MRSA	2008
GCA_000641515.1	48	Netherlands	Hominine	MRSA	2008
GCA_000641535.1	48	Netherlands	Hominine	MRSA	2008
GCA_000641555.1	48	Netherlands	Hominine	MRSA	2008
GCA_000641575.1	48	Netherlands	Hominine	MRSA	2008
GCA_000641595.1	48	Netherlands	Hominine	MRSA	2008
GCA_000641635.1	48	Netherlands	Hominine	MRSA	2008
GCA_000641695.1	44	Netherlands	Hominine	MRSA	2008
GCA_000641715.1	48	Netherlands	Hominine	MRSA	2008
GCA_000641755.1	48	Netherlands	Hominine	MRSA	2008
GCA_000641815.1	48	Netherlands	Hominine	MRSA	2008
GCA_000641875.1	44	Netherlands	Hominine	MRSA	2008
GCA_000641895.1	48	Netherlands	Hominine	MRSA	2008
GCA_000641915.1	44	Netherlands	Hominine	MRSA	2008
GCA_000641955.1	48	Netherlands	Hominine	MRSA	2008
GCA_000641975.1	48	Netherlands	Hominine	MRSA	2008
GCA_000641995.1	44	Netherlands	Hominine	MRSA	2008
GCA_000642015.1	48	Netherlands	Hominine	MRSA	2008
GCA_000642035.1	48	Netherlands	Hominine	MRSA	2008
GCA_000642055.1	48	Netherlands	Hominine	MRSA	2008
GCA_000642075.1	48	Netherlands	Hominine	MRSA	2008
GCA_000642115.1	48	Netherlands	Hominine	MRSA	2008
GCA_000642135.1	47	Netherlands	Hominine	MRSA	2008
GCA_000642155.1	44	Netherlands	Hominine	MRSA	2008
GCA_000642175.1	48	Netherlands	Hominine	MRSA	2008
GCA_000642195.1	44	Netherlands	Hominine	MRSA	2008
GCA_000642215.1	42	Netherlands	Hominine	MRSA	2008
GCA_000642235.1	48	Netherlands	Hominine	MRSA	2008
GCA_000642255.1	44	Netherlands	Hominine	MRSA	2008
GCA_000642275.1	44	Netherlands	Hominine	MRSA	2008
GCA_000642315.1	44	Netherlands	Hominine	MRSA	2008
GCA_000642335.1	48	Netherlands	Hominine	MRSA	2008
GCA_000642395.1	48	Netherlands	Hominine	MRSA	2008
GCA_000642435.1	48	Netherlands	Hominine	MRSA	2008
GCA_000642455.1	48	Netherlands	Hominine	MRSA	2008
GCA_000642475.1	44	Netherlands	Hominine	MRSA	2008
GCA_000642535.1	44	Netherlands	Hominine	MRSA	2009
GCA_000642575.1	44	Netherlands	Hominine	MRSA	2009
GCA_000642615.1	48	Netherlands	Hominine	MRSA	2009
GCA_000642675.1	48	Netherlands	Hominine	MRSA	2009
GCA_000642755.1	48	Netherlands	Hominine	MRSA	2009
GCA_000642795.1	48	Netherlands	Hominine	MRSA	2009
GCA_000642815.1	47	Netherlands	Hominine	MRSA	2009
GCA_000642835.1	48	Netherlands	Hominine	MRSA	2009
GCA_000642855.1	48	Netherlands	Hominine	MRSA	2009
GCA_000642895.1	44	Netherlands	Hominine	MRSA	2009
GCA_000642915.1	44	Netherlands	Hominine	MRSA	2009
GCA_000642955.1	48	Netherlands	Hominine	MRSA	2009
GCA_000643015.1	44	Netherlands	Hominine	MRSA	2009
GCA_000643035.1	44	Netherlands	Hominine	MRSA	2009
GCA_000643055.1	48	Netherlands	Hominine	MRSA	2009
GCA_000643095.1	44	Netherlands	Hominine	MRSA	2009
GCA_000643115.1	44	Netherlands	Hominine	MRSA	2009
GCA_000643135.1	44	Netherlands	Hominine	MRSA	2009
GCA_000643155.1	44	Netherlands	Hominine	MRSA	2009
GCA_000643215.1	44	Netherlands	Hominine	MRSA	2009
GCA_000643255.1	44	Netherlands	Hominine	MRSA	2009
GCA_000643275.1	44	Netherlands	Hominine	MRSA	2009
GCA_000643315.1	44	Netherlands	Hominine	MRSA	2009
GCA_000643335.1	44	Netherlands	Hominine	MRSA	2009
GCA_000643415.1	44	Netherlands	Hominine	MRSA	2009
GCA_000643455.1	42	Netherlands	Hominine	MRSA	2009
GCA_000643475.1	48	Netherlands	Hominine	MRSA	2009
GCA_000643515.1	48	Netherlands	Hominine	MRSA	2009

GCA_000643535.1	47	Netherlands	Hominine	MRSA	2009
GCA_000643555.1	44	Netherlands	Hominine	MRSA	2009
GCA_000643575.1	44	Netherlands	Hominine	MRSA	2009
GCA_000643595.1	48	Netherlands	Hominine	MRSA	2009
GCA_000643615.1	48	Netherlands	Hominine	MRSA	2009
GCA_000643635.1	48	Netherlands	Hominine	MRSA	2009
GCA_000643655.1	48	Netherlands	Hominine	MRSA	2009
GCA_000643675.1	48	Netherlands	Hominine	MRSA	2009
GCA_000643715.1	44	Netherlands	Hominine	MRSA	2009
GCA_000643755.1	48	Netherlands	Hominine	MRSA	2009
GCA_000643795.1	48	Netherlands	Hominine	MRSA	2009
GCA_000643815.1	48	Netherlands	Hominine	MRSA	2009
GCA_000643835.1	48	Netherlands	Hominine	MRSA	2009
GCA_000643855.1	48	Netherlands	Hominine	MRSA	2009
GCA_000643895.1	48	Netherlands	Hominine	MRSA	2009
GCA_000643915.1	48	Netherlands	Hominine	MRSA	2009
GCA_000643935.1	44	Netherlands	Hominine	MRSA	2009
GCA_000643955.1	48	Netherlands	Hominine	MRSA	2009
GCA_000643975.1	48	Netherlands	Hominine	MRSA	2009
GCA_000643995.1	48	Netherlands	Hominine	MRSA	2009
GCA_000644015.1	48	Netherlands	Hominine	MRSA	2009
GCA_000644035.1	48	Netherlands	Hominine	MRSA	2009
GCA_000644075.1	48	Netherlands	Hominine	MRSA	2009
GCA_000644115.1	48	Netherlands	Hominine	MRSA	2009
GCA_000644135.1	48	Netherlands	Hominine	MRSA	2009
GCA_000644175.1	48	Netherlands	Hominine	MRSA	2009
GCA_000644215.1	44	Netherlands	Hominine	MRSA	2009
GCA_000644235.1	48	Netherlands	Hominine	MRSA	2009
GCA_000644255.1	44	Netherlands	Hominine	MRSA	2009
GCA_000644275.1	44	Netherlands	Hominine	MRSA	2009
GCA_000644295.1	44	Netherlands	Hominine	MRSA	2009
GCA_000644315.1	44	Netherlands	Hominine	MRSA	2009
GCA_000644335.1	47	Netherlands	Hominine	MRSA	2009
GCA_000644375.1	48	Netherlands	Hominine	MRSA	2009
GCA_000644395.1	48	Netherlands	Hominine	MRSA	2009
GCA_000644415.1	48	Netherlands	Hominine	MRSA	2009
GCA_000644455.1	48	Netherlands	Hominine	MRSA	2009
GCA_000644495.1	48	Netherlands	Hominine	MRSA	2009
GCA_000644515.1	48	Netherlands	Hominine	MRSA	2009
GCA_000644535.1	48	Netherlands	Hominine	MRSA	2009
GCA_000644555.1	48	Netherlands	Hominine	MRSA	2009
GCA_000644575.1	48	Netherlands	Hominine	MRSA	2009
GCA_000644595.1	44	Netherlands	Hominine	MRSA	2009
GCA_000644615.1	44	Netherlands	Hominine	MRSA	2009
GCA_000644635.1	44	Netherlands	Hominine	MRSA	2009
GCA_000644655.1	48	Netherlands	Hominine	MRSA	2009
GCA_000644695.1	48	Netherlands	Hominine	MRSA	2009
GCA_000644715.1	48	Netherlands	Hominine	MRSA	2009
GCA_000644735.1	48	Netherlands	Hominine	MRSA	2009
GCA_000644755.1	48	Netherlands	Hominine	MRSA	2009
GCA_000644775.1	48	Netherlands	Hominine	MRSA	2009
GCA_000644815.1	48	Netherlands	Hominine	MRSA	2009
GCA_000644855.1	44	Netherlands	Hominine	MRSA	2009
GCA_000644875.1	48	Netherlands	Hominine	MRSA	2009
GCA_000644895.1	48	Netherlands	Hominine	MRSA	2009
GCA_000644915.1	44	Netherlands	Hominine	MRSA	2009
GCA_000644935.1	48	Netherlands	Hominine	MRSA	2009
GCA_000644955.1	48	Netherlands	Hominine	MRSA	2009
GCA_000644975.1	44	Netherlands	Hominine	MRSA	2009
GCA_000644995.1	48	Netherlands	Hominine	MRSA	2009
GCA_000645015.1	48	Netherlands	Hominine	MRSA	2009
GCA_000645035.1	48	Netherlands	Hominine	MRSA	2009
GCA_000645055.1	48	Netherlands	Hominine	MRSA	2009
GCA_000645075.1	48	Netherlands	Hominine	MRSA	2009
GCA_000645095.1	48	Netherlands	Hominine	MRSA	2009
GCA_000645135.1	48	Netherlands	Hominine	MRSA	2009
GCA_000645155.1	48	Netherlands	Hominine	MRSA	2009
GCA_000645195.1	48	Netherlands	Hominine	MRSA	2009
GCA_000645235.1	48	Netherlands	Hominine	MRSA	2009
GCA_000645255.1	48	Netherlands	Hominine	MRSA	2009
GCA_000645275.1	48	Netherlands	Hominine	MRSA	2009
GCA_000645295.1	48	Netherlands	Hominine	MSSA	2008

GCA_000645315.1	48	Netherlands	Hominine	MRSA	2008
GCA_000645355.1	52	Netherlands	Hominine	MSSA	2008
GCA_000645375.1	47	Netherlands	Hominine	MSSA	2008
GCA_000645495.1	47	Netherlands	Hominine	MSSA	2008
GCA_000645535.1	52	Netherlands	Hominine	MSSA	2009
GCA_000645555.1	52	Netherlands	Hominine	MSSA	2009
GCA_000645655.1	47	Netherlands	Hominine	MSSA	2009
GCA_000645675.1	47	Netherlands	Hominine	MSSA	2009
GCA_000645715.1	55	Netherlands	Hominine	MSSA	2009
GCA_000645755.1	44	Netherlands	Hominine	MSSA	2009
GCA_000645775.1	47	Netherlands	Hominine	MSSA	2009
GCA_000645795.1	40	Netherlands	Hominine	MSSA	2009
GCA_000645815.1	47	Netherlands	Hominine	MSSA	2009
GCA_000645855.1	47	Netherlands	Hominine	MSSA	2009
GCA_000645895.1	40	Netherlands	Hominine	MSSA	2009
GCA_000645935.1	42	Netherlands	Hominine	MSSA	2009
GCA_000645975.1	47	Netherlands	Hominine	MSSA	2009
GCA_000646055.1	47	Netherlands	Hominine	MSSA	2009
GCA_000646115.1	47	Netherlands	Hominine	MSSA	2009
GCA_000646135.1	47	Netherlands	Hominine	MSSA	2009
GCA_000646155.1	40	Netherlands	Hominine	MSSA	2009
GCA_000646195.1	48	Netherlands	Hominine	MRSA	2011
GCA_000646215.1	48	Netherlands	Hominine	MRSA	2011
GCA_000646235.1	48	Netherlands	Hominine	MRSA	2011
GCA_000646255.1	48	Netherlands	Hominine	MRSA	2011
GCA_000646295.1	48	Netherlands	Hominine	MRSA	2011
GCA_000646315.1	44	Netherlands	Hominine	MRSA	2011
GCA_000646335.1	44	Netherlands	Hominine	MRSA	2011
GCA_000646355.1	44	Netherlands	Hominine	MRSA	2011
GCA_000646415.1	48	Netherlands	Hominine	MRSA	2011
GCA_000646435.1	47	Netherlands	Hominine	MRSA	2011
GCA_000646455.1	47	Netherlands	Hominine	MRSA	2011
GCA_000646475.1	48	Netherlands	Hominine	MRSA	2011
GCA_000646495.1	48	Netherlands	Hominine	MRSA	2011
GCA_000646515.1	48	Netherlands	Hominine	MRSA	2011
GCA_000646535.1	48	Netherlands	Hominine	MRSA	2011
GCA_000646555.1	47	Netherlands	Hominine	MRSA	2011
GCA_000646575.1	48	Netherlands	Hominine	MRSA	2011
GCA_000646595.1	48	Netherlands	Hominine	MRSA	2011
GCA_000646615.1	48	Netherlands	Hominine	MRSA	2011
GCA_000646635.1	48	Netherlands	Hominine	MRSA	2011
GCA_000646655.1	48	Netherlands	Hominine	MRSA	2011
GCA_000646675.1	48	Netherlands	Hominine	MRSA	2011
GCA_000646695.1	48	Netherlands	Hominine	MRSA	2011
GCA_000646735.1	48	Netherlands	Hominine	MRSA	2011
GCA_000646755.1	48	Netherlands	Hominine	MRSA	2011
GCA_000646775.1	47	Netherlands	Hominine	MRSA	2011
GCA_000646795.1	47	Netherlands	Hominine	MRSA	2011
GCA_000646815.1	48	Netherlands	Hominine	MRSA	2011
GCA_000646855.1	44	Netherlands	Hominine	MRSA	2011
GCA_000646875.1	48	Netherlands	Hominine	MRSA	2011
GCA_000646895.1	47	Netherlands	Hominine	MRSA	2011
GCA_000646915.1	44	Netherlands	Hominine	MRSA	2011
GCA_000646935.1	48	Netherlands	Hominine	MRSA	2011
GCA_000646955.1	48	Netherlands	Hominine	MRSA	2011
GCA_000646995.1	48	Netherlands	Hominine	MRSA	2011
GCA_000647015.1	48	Netherlands	Hominine	MRSA	2011
GCA_000647035.1	48	Netherlands	Hominine	MRSA	2011
GCA_000647055.1	44	Netherlands	Hominine	MRSA	2011
GCA_000647075.1	48	Netherlands	Hominine	MRSA	2011
GCA_000647095.1	48	Netherlands	Hominine	MRSA	2011
GCA_000647115.1	48	Netherlands	Hominine	MRSA	2011
GCA_000647135.1	48	Netherlands	Hominine	MRSA	2011
GCA_000647155.1	48	Netherlands	Hominine	MRSA	2011
GCA_000647175.1	47	Netherlands	Hominine	MSSA	2011
GCA_000647215.1	48	Netherlands	Hominine	MSSA	2011
GCA_000647235.1	49	Netherlands	Hominine	MSSA	2011
GCA_000647275.1	47	Netherlands	Hominine	MSSA	2011
GCA_000682855.1	48	Netherlands	Hominine	MSSA	2011
GCA_000682875.1	48	Netherlands	Hominine	MRSA	2011
GCA_000682895.1	48	Netherlands	Hominine	MRSA	2011
GCA_000682915.1	48	Netherlands	Hominine	MRSA	2011

GCA_000682935.1	48	Netherlands	Hominine	MRSA	2011
GCA_000682955.1	48	Netherlands	Hominine	MRSA	2011
GCA_000682975.1	44	Netherlands	Hominine	MRSA	2011
GCA_000682995.1	48	Netherlands	Hominine	MRSA	2011
GCA_000683015.1	47	Netherlands	Hominine	MRSA	2011
GCA_000683035.1	48	Netherlands	Hominine	MRSA	2011
GCA_000683055.1	48	Netherlands	Hominine	MRSA	2011
GCA_000683075.1	42	Netherlands	Hominine	MRSA	2009
GCA_000683095.1	47	Netherlands	Hominine	MSSA	2009
GCA_000683115.1	44	Netherlands	Hominine	MSSA	2009
GCA_000683175.1	47	Netherlands	Hominine	MSSA	2009
GCA_000683195.1	48	Netherlands	Hominine	MSSA	2008
GCA_000683215.1	47	Netherlands	Hominine	MSSA	2008
GCA_000683235.1	48	Netherlands	Hominine	MSSA	2008
GCA_000683255.1	48	Netherlands	Hominine	MRSA	2009
GCA_000683295.1	48	Netherlands	Hominine	MRSA	2009
GCA_000683315.1	44	Netherlands	Hominine	MRSA	2009
GCA_000683335.1	44	Netherlands	Hominine	MRSA	2009
GCA_000683355.1	44	Netherlands	Hominine	MRSA	2009
GCA_000683375.1	48	Netherlands	Hominine	MRSA	2009
GCA_000683435.1	44	Netherlands	Hominine	MRSA	2009
GCA_000683475.1	48	Netherlands	Hominine	MRSA	2009
GCA_000683495.1	44	Netherlands	Hominine	MRSA	2009
GCA_000683535.1	44	Netherlands	Hominine	MRSA	2009
GCA_000683555.1	53	Netherlands	Hominine	MRSA	2009
GCA_000683575.1	44	Netherlands	Hominine	MRSA	2009
GCA_000683595.1	48	Netherlands	Hominine	MRSA	2009
GCA_000683615.1	48	Netherlands	Hominine	MRSA	2009
GCA_000683675.1	48	Netherlands	Hominine	MRSA	2009
GCA_000683715.1	48	Netherlands	Hominine	MRSA	2009
GCA_000683735.1	48	Netherlands	Hominine	MRSA	2009
GCA_000683775.1	48	Netherlands	Hominine	MRSA	2009
GCA_000683795.1	48	Netherlands	Hominine	MRSA	2008
GCA_000683835.1	48	Netherlands	Hominine	MRSA	2008
GCA_000683855.1	48	Netherlands	Hominine	MRSA	2008
GCA_000683875.1	44	Netherlands	Hominine	MRSA	2008
GCA_000683895.1	48	Netherlands	Hominine	MRSA	2008
GCA_000683915.1	47	Netherlands	Hominine	MRSA	2008
GCA_000683955.1	44	Netherlands	Hominine	MRSA	2008
GCA_000683975.1	42	Netherlands	Hominine	MRSA	2008
GCA_000683995.1	44	Netherlands	Hominine	MRSA	2008
GCA_000684015.1	44	Netherlands	Hominine	MRSA	2008
GCA_000684035.1	48	Netherlands	Hominine	MRSA	2008
GCA_000684075.1	44	Netherlands	Hominine	MRSA	2008
GCA_000684095.1	48	Netherlands	Hominine	MRSA	2008
GCA_000684155.1	44	Netherlands	Hominine	MRSA	2008
GCA_000684175.1	44	Netherlands	Hominine	MRSA	2008
GCA_000684195.1	48	Netherlands	Hominine	MRSA	2009
GCA_000684235.1	44	Netherlands	Hominine	MRSA	2008
GCA_000684255.1	44	Netherlands	Hominine	MRSA	2008
GCA_000684275.1	44	Netherlands	Hominine	MRSA	2008
GCA_000684335.1	48	Germany	Meleagrine	MRSA	2009
GCA_000684355.1	48	Germany	Meleagrine	MRSA	2009
GCA_000684375.1	48	Belgium	Galline	MRSA	2006
GCA_000684495.1	48	Netherlands	Equine	MRSA	2010
GCA_000684515.1	48	Netherlands	Ovine	MRSA	2010
GCA_000684535.1	48	Spain	Hominine	MRSA	2009
GCA_000684715.1	47	Germany	Galline	MRSA	2011
GCA_000684795.1	47	Netherlands	Hominine	MRSA	2010
GCA_001078665.1	14	China	Environmental	MSSA	2014
GCA_001455955.1	47	Brazil	Porcine	MRSA	2012
GCA_001465635.1	44	Netherlands	Hominine	MRSA	2009
GCA_001465675.1	48	Netherlands	Hominine	MRSA	2008
GCA_001465755.1	45	Netherlands	Hominine	MRSA	2008
GCA_001696355.1	5	France	Hominine	MSSA	2012
GCA_001696365.1	5	France	Hominine	MSSA	2010
GCA_001696415.1	5	France	Hominine	MSSA	2014
GCA_001696445.1	5	France	Hominine	MSSA	2015
GCA_001726145.1	47	Brazil	Hominine	MRSA	2010
GCA_001887075.1	40	Canada	Galline	MRSA	2013
GCA_001940425.1	47	Denmark	Hominine	MRSA	2007
GCA_002025125.1	48	Germany	Porcine	MRSA	2012

GCA_002089035.2	5	Canada	Hominine	MSSA	2014
GCA_002099175.1	48	Denmark	Bovine	MRSA	2016
GCA_002204575.1	40	USA	Porcine	MRSA	2010
GCA_002204735.1	47	Switzerland	Porcine	MRSA	2009
GCA_002208335.1	5	France	Hominine	MSSA	2013
GCA_002208395.1	5	France	Hominine	MSSA	2010
GCA_002208415.1	47	France	Hominine	MRSA	2010
GCA_002208435.1	5	France	Hominine	MSSA	2014
GCA_002208455.1	5	France	Hominine	MSSA	2012
GCA_002208475.1	40	France	Hominine	MSSA	2014
GCA_002208535.1	5	France	Hominine	MSSA	2011
GCA_002208555.1	5	France	Hominine	MSSA	2014
GCA_002250725.1	5	USA	Hominine	MSSA	2014
GCA_002307435.1	21	China	Hominine	MRSA	2014
GCA_002407225.1	21	China	Hominine	MRSA	2014
GCA_002723755.1	21	China	Hominine	MRSA	2015
GCA_002934925.1	48	South Africa	Porcine	MRSA	2016
GCA_002934945.1	48	South Africa	Porcine	MRSA	2016
GCA_003001175.1	48	Germany	Equine	MRSA	2014
GCA_003001185.1	48	Germany	Equine	MRSA	2014
GCA_003001215.1	48	Germany	Equine	MRSA	2014
GCA_003001225.1	48	Germany	Equine	MRSA	2014
GCA_003001255.1	48	Germany	Equine	MRSA	2014
GCA_003001275.1	48	Germany	Equine	MRSA	2014
GCA_003001295.1	48	Germany	Equine	MRSA	2014
GCA_003001315.1	48	Germany	Equine	MRSA	2014
GCA_003001335.1	48	Germany	Equine	MRSA	2014
GCA_003001345.1	48	Germany	Equine	MRSA	2015
GCA_003001375.1	48	Germany	Equine	MRSA	2015
GCA_003001395.1	48	Germany	Equine	MRSA	2015
GCA_003001415.1	48	Germany	Equine	MRSA	2015
GCA_003001435.1	48	Germany	Equine	MRSA	2015
GCA_003001455.1	48	Germany	Equine	MRSA	2015
GCA_003001475.1	48	Germany	Equine	MRSA	2014
GCA_003001485.1	48	Germany	Equine	MRSA	2015
GCA_003057555.1	48	Germany	Equine	MRSA	2015
GCA_003237125.1	47	Italy	Hominine	MSSA	2014
GCA_003238765.1	48	Italy	Hominine	MRSA	2014
GCA_003309235.1	12	China	Porcine	MSSA	2016
GCA_003309285.1	10	China	Porcine	MRSA	2016
GCA_003309325.1	10	China	Porcine	MSSA	2016
GCA_003309355.1	40	China	Porcine	MSSA	2016
GCA_003309445.1	40	China	Porcine	MSSA	2016
GCA_003309465.1	40	China	Porcine	MSSA	2016
GCA_003309525.1	10	China	Hominine	MSSA	2015
GCA_003309545.1	10	China	Hominine	MSSA	2015
GCA_003309665.1	14	China	Hominine	MSSA	2015
GCA_003309805.1	10	China	Porcine	MRSA	2016
GCA_003309845.1	10	China	Porcine	MSSA	2016
GCA_003309865.1	47	China	Porcine	MSSA	2016
GCA_003309885.1	10	China	Porcine	MSSA	2016
GCA_003309905.1	10	China	Porcine	MSSA	2016
GCA_003309925.1	12	China	Hominine	MSSA	2015
GCA_003327835.1	48	Germany	Hominine	MRSA	2010
GCA_003327875.1	48	Germany	Hominine	MSSA	2012
GCA_003327885.1	48	Germany	Equine	MRSA	2014
GCA_003327935.1	47	Germany	Porcine	MRSA	2016
GCA_003327955.1	48	Germany	Hominine	MSSA	2015
GCA_003327965.1	48	Germany	Hominine	MRSA	2012
GCA_003327995.1	47	Germany	Porcine	MSSA	2016
GCA_003328005.1	48	Germany	Hominine	MRSA	2011
GCA_003328335.1	48	Germany	Equine	MRSA	2016
GCA_003328355.1	48	Germany	Equine	MRSA	2016
GCA_003328375.1	48	Germany	Equine	MRSA	2016
GCA_003328385.1	48	Germany	Equine	MRSA	2016
GCA_003328395.1	48	Germany	Equine	MRSA	2016
GCA_003328405.1	48	Germany	Equine	MRSA	2016
GCA_003353135.1	51	USA	Ovine	MSSA	2018
GCA_003353145.1	51	USA	Ovine	MSSA	2018
GCA_003353195.1	51	USA	Ovine	MSSA	2018
GCA_003353235.1	51	USA	Ovine	MSSA	2018
GCA_003353265.1	51	USA	Ovine	MSSA	2018

GCA_003581515.1	47	Czech Republic	Hominine	MRSA	2017
GCA_003583795.1	47	Germany	Poultry	MRSA	2017
GCA_003583815.1	47	Poland	Poultry	MRSA	2017
GCA_003697325.1	21	China	Hominine	MRSA	2015
GCA_003720975.1	5	USA	Hominine	MSSA	2018
GCA_004011165.1	47	South Korea	Porcine	MRSA	2017
GCA_004012035.1	47	South Korea	Hominine	MRSA	2017
GCA_004681195.1	47	South Korea	Porcine	MRSA	2017
GCA_005931015.1	1	China	Hominine	MSSA	2014
GCA_007726325.1	56	USA	Meleagrine	MSSA	2010
GCA_900088495.1	48	Finland	Porcine	MRSA	2015
GCA_900088505.1	48	Finland	Porcine	MRSA	2015
GCA_900088515.1	47	Finland	Porcine	MRSA	2015
GCA_900250545.1	47	Denmark	Hominine	MRSA	2014
GCA_900250695.1	47	Denmark	Hominine	MRSA	2014
GCA_900250715.1	47	Denmark	Hominine	MRSA	2014
GCA_900250965.1	47	Denmark	Hominine	MRSA	2014
GCA_900251395.1	47	Denmark	Hominine	MRSA	2014
GCA_900251735.1	47	Denmark	Hominine	MRSA	2014
GCA_900251815.1	47	Denmark	Hominine	MRSA	2014
GCA_900251835.1	47	Denmark	Hominine	MRSA	2014
GCA_900251945.1	47	Denmark	Hominine	MRSA	2014
GCA_900252185.1	48	Denmark	Hominine	MRSA	2014
GCA_900324215.1	47	Germany	Hominine	MRSA	2008
GCA_900324235.1	47	Germany	Hominine	MRSA	2010
GCA_900324265.1	47	Germany	Hominine	MRSA	2011
GCA_900324315.1	48	Germany	Hominine	MRSA	2011
GCA_900324335.1	48	Germany	Hominine	MRSA	2012
GCA_900324345.1	48	Germany	Hominine	MRSA	2012
GCA_900324415.1	48	Germany	Hominine	MRSA	2015
GCA_900409475.1	47	Germany	Hominine	MRSA	2011
JL108	15	China	Hominine	MSSA	2018
JL113	34	China	Hominine	MSSA	2018
JL116	15	China	Hominine	MSSA	2018
JL28	21	China	Hominine	MRSA	2018
JL42	21	China	Hominine	MSSA	2018
JL69	28	China	Hominine	MSSA	2018
JL73	20	China	Hominine	MSSA	2018
JL84	15	China	Hominine	MSSA	2018
S82	21	China	Hominine	MRSA	2018
S85	21	China	Hominine	MRSA	2018
SRR5054902	30	China	Hominine	MSSA	2010
SRR5054904	15	China	Hominine	MSSA	2014
SRR5054907	38	China	Hominine	MSSA	2011
SRR5054908	15	China	Hominine	MSSA	2012
SRR5054909	15	China	Hominine	MSSA	2014
SRR5054910	10	China	Hominine	MSSA	2012
SRR5054912	27	China	Hominine	MSSA	2010
SRR5054913	39	China	Hominine	MSSA	2012
SRR5054914	39	China	Hominine	MSSA	2014
SRR5054915	15	China	Hominine	MSSA	2012
SRR5054916	23	China	Hominine	MSSA	2012
SRR5054917	23	China	Hominine	MRSA	2012
SRR5054918	15	China	Hominine	MSSA	2014
SRR5054920	39	China	Hominine	MSSA	2012
SRR5054922	14	China	Hominine	MSSA	2012
SRR5054924	15	China	Hominine	MSSA	2012
SRR5054925	21	China	Hominine	MRSA	2014
SRR5054926	15	China	Hominine	MSSA	2011
SRR5054927	14	China	Hominine	MSSA	2014
SRR5054929	21	China	Hominine	MSSA	2010
SRR5054931	11	China	Hominine	MSSA	2010
SRR5054932	36	China	Hominine	MSSA	2011
SRR5054935	37	China	Hominine	MSSA	2010
SRR5054936	21	China	Hominine	MRSA	2014
SRR5054938	21	China	Hominine	MSSA	2011
SRR5054939	10	China	Hominine	MSSA	2012
SRR5054940	2	China	Hominine	MSSA	2014
SRR5054942	10	China	Hominine	MSSA	2012
SRR5054948	15	China	Hominine	MSSA	2014
SRR5054950	35	China	Hominine	MSSA	2010
SRR5054951	39	China	Hominine	MSSA	2011

SRR5054952	28	China	Hominine	MSSA	2012
SRR5054954	15	China	Hominine	MSSA	2014
SRR5054956	15	China	Hominine	MSSA	2011
SRR5054962	3	China	Hominine	MSSA	2014
SRR5054966	36	China	Hominine	MSSA	2012
SRR5054967	15	China	Hominine	MSSA	2010
SRR5054969	39	China	Hominine	MSSA	2014
SRR5054970	14	China	Hominine	MSSA	2011
SRR5054973	21	China	Hominine	MRSA	2012
SRR5054976	15	China	Hominine	MSSA	2012
SRR5054977	3	China	Hominine	MSSA	2012

NA: not available

ND: not detected

Table S3 The list of 194 VFs

VF_ID	Uniprot access No. or GenBank access No. or the GenBank access No. of the isolates carried the VFs	length	Refrence (doi)
aaa	AJ250906.1	1060	10.1128/IAI.73.8.4793–4802.2005
arlR	AAF85896.1	660	10.1080/22221751.2019.1595984
arlS	AAF85897.1	1356	10.1080/22221751.2019.1595984
atl	NC_007795.1_964093-967863	3771	10.3389/fcimb.2016.00051
aucA	AAN71834.1	156	10.1128/JB.06203-11
bacA	AF228662.1	876	10.1099/00221287-146-7-1547
bap	AY220730.1_5366-12196	6831	10.1128/JB.183.9.2888–2896.2001
bbp	BAE97666.1	3468	10.1038/nrmicro3161
bsaA1	BAB95631.1	144	10.1128/JB.01375-09
bsaA2	BAB95630.1	144	10.1128/JB.01375-09
cggr	AUS75299.1	1014	10.1186/s12864-017-3516-x
chp	BAF68149.1	450	10.1186/s12864-017-3516-x
clfA	CP000255.1_859148-861949	2802	10.1038/nrmicro3161
clfB	CP000255.1_2774340-2777039	2700	10.1038/nrmicro3161
clpA	AAA23583.1	2277	10.1016/j.resmic.2009.08.006
clpB	NC_007795.1_882636-885245	2610	10.1016/j.resmic.2009.08.006
clpC	NC_007795.1_505226-507682	2457	10.1016/j.resmic.2009.08.006
clpE	CAB13243.1	2100	10.1016/j.resmic.2009.08.006
clpL	CP000255.1_2685195-2687300	2106	10.1016/j.resmic.2009.08.006
clpP	CP000255.1_838576-839163	588	10.1016/j.resmic.2009.08.006
clpQ	NC_007795.1_1177666-1178211	546	10.1016/j.resmic.2009.08.006
clpX	NC_007795.1_1677874-1679136	1263	10.1016/j.resmic.2009.08.006
clpY	BAB57416.1	1404	10.1016/j.resmic.2009.08.006
cna	AAA20874.1	3552	10.1128/mBio.01529-16.
cna_1	AB266875.1	2430	NA
coa_type1	AB158549.1_2462-4522	2061	10.1016/j.mimet.2008.07.003
coa_type2	BA000018.3_264676-266652	1977	10.1016/j.mimet.2008.07.003
coa_type3	CP000253.1_211732-213642	1911	10.1016/j.mimet.2008.07.003
coa_type4	AB158550.1_2462-4453	1991	10.1016/j.mimet.2008.07.003
coa_type5	AB158551.1_2462-4639	2178	10.1016/j.mimet.2008.07.003
coa_type6a	AB158552.1_2462-4450	1989	10.1016/j.mimet.2008.07.003
coa_type6b	AB373752.1	1884	10.1016/j.mimet.2008.07.003
coa_type6c	AB373755.1	2151	10.1016/j.mimet.2008.07.003
coa_type7	BA000033.2_242721-244622	1902	10.1016/j.mimet.2008.07.003
coa_type8	AB158553.1_2462-4741	2280	10.1016/j.mimet.2008.07.003
crtM	CP000255.1_2703659-2704522	864	10.1128/AEM.01998-19
crtN	CP000255.1_2702139-2703647	1509	10.1128/AEM.01998-19
crtO	CP000255.1_2707178-2707666	489	10.1128/AEM.01998-19
dltA	NC_002745.2_898448-899905	1458	10.1128/AAC.00437-10
dltB	NC_002745.2_899902-901116	1215	10.1128/AAC.00437-10
dltC	NC_002745.2_901134-901370	237	10.1128/AAC.00437-10
dltD	NC_002745.2_901367-902542	1176	10.1128/AAC.00437-10
eap	NZ_CP023390.1_2085907-2087661	1755	10.1128/JCM.01425-07
ebh	NZ_CP027476.1_1456811-1488076	31266	10.1371/journal.ppat.1003819
ebpS	AAC44135.2	1461	10.1074/jbc.M107621200
edin-A	M63917.1_80-823	744	10.1086/322983
edin-B	BAC22946.1	744	10.1086/322983
edin-C	AP003088.1_c2079-1336	744	10.1086/322983
efb	NZ_CP023390.1_1172925-1173422	498	10.1128/IAI.01423-09
emp	NZ_CP023390.1_854656-855678	1023	10.1099/mic.0.27902-0
eno	NC_007795.1_782057-783361	1305	10.1107/S1399004715018830
est	CXN83422.1	741	10.1093/jac/dkn363
eta	AAA26626.1	843	10.3390/toxins2051148
etb	AAA26628.1	834	10.3390/toxins2051148
etc	AB036769.1_160-996	837	10.1128/IAI.70.10.5835–5845.2002
etd	AB057421.1_5409-6254	846	10.1128/IAI.70.10.5835–5845.2002
ete	CP025395.1_2272706-2273548	843	10.1038/s41598-019-52777-3
exhA	AAN32970.1	822	10.3390/toxins2051148
exhB	AAN32971.1	906	10.3390/toxins2051148
exhC	AAN32972.1	837	10.3390/toxins2051148
exhD	AAN32973.1	819	10.3390/toxins2051148
exhI	BAI49625.1	831	10.1111/j.1574-6968.2009.01823.x
FLIPr	NC_002745.2_1133925-1134326	402	10.4049/jimmunol.177.11.8017

FLIPr-like	NC_003923.1_1135148-1135546	399	10.4049/jimmunol.0801523
gapA1	CAG39837.1	1011	10.1186/s12864-017-3516-x
gpmI	BAB56937.1	1518	10.1371/journal.ppat.1007971
hlb	AAW36972.1	993	10.1128/MMBR.00082-15.
hld	BAB58197.1	135	10.1016/j.peptides.2008.12.017
hlgA	BAB96207.1	930	10.1021/ci050175y
hlgB	BAB96209.1	978	10.1021/ci050175y
hlgC	BAB96208.1	948	10.1021/ci050175y
hly	CAA25801.1	960	10.3390/toxins5061140.
hysA	CP000255.1_2338092-2340515	2424	10.1128/IAI.01710-14
icaA	AF086783.1_2330-3568	1169	10.3205/dgkh000203
icaB	AF086783.1_3834-4706	873	10.3205/dgkh000203
icaC	AF086783.1_4693-5745	1053	10.3205/dgkh000203
icaD	AF086783.1_3532-3837	306	10.3205/dgkh000203
icaR	AF086783.1_c2166-1606	561	10.3389/fcimb.2012.00038
isdC	CP000255.1_1125752-1126435	684	10.1038/nrmicro3161
katA	NC_002745.2_1332093-1333616	1524	10.1007/s00203-014-1048-1
lexA	CAG40348.1	624	10.1186/s12864-017-3516-x
lip1	CP000253.1_c2779036-2781078	2043	10.1128/JB.00064-12
lip2	CP000253.1_314326-316398	2073	10.1128/JB.00064-12
lukA(G)	CP000255.1_2129806-2130861	1056	10.1128/MMBR.00055-13
lukB(H)	CP000255.1_2128768-2129784	1017	10.1128/MMBR.00055-13
lukD	CAA73668.1	984	10.1111/j.1348-0421.2003.tb02789.x
lukE	CAA73667.1	945	10.1111/j.1348-0421.2003.tb02789.x
lukF	AB006796.1	978	10.1128/MMBR.00055-13
lukF-	NC_017337.1_1975486-1976454	969	10.1128/MMBR.00055-13
lukM	NC_017337.1_1976456-1977382	927	10.1128/MMBR.00055-13
lukP	SIO73073.1	936	10.1038/srep40660
lukQ	SIO73072.1	981	10.1038/srep40660
lukS	AB006796.1	939	10.1128/MMBR.00055-13
mgrA	CP000255.1_746323-746766	444	10.1128/microbiolspec.GPP3-0031-2018
mprF	NC_002745.2_1363612-1366134	2523	10.1128/AAC.00437-10
nuc	BX571856.1_895661-896347	687	10.1007/s00284-017-1274-2
oatA	NC_002745.2_c2645038-2646849	1812	10.1111/2049-632X.12099
pgk	BAB41961.1	1191	10.1186/s12864-017-3516-x
pls	AF115379.2	4914	10.1038/nrmicro3161
psm-beta1	AFD54319.1	135	10.1111/1574-6976.12057.
psm-beta2	AFD54320.1	135	10.1111/1574-6976.12057.
psm-mec	BAJ06379.1	69	10.1111/1574-6976.12057.
psmA1	A9JX05	66	10.1111/1574-6976.12057.
psmA2	A9JX06	66	10.1111/1574-6976.12057.
psmA3	A9JX07	69	10.1111/1574-6976.12057.
psmA4	A9JX08	63	10.1111/1574-6976.12057.
rnr	BAB56942.2	2373	10.2217/fmb.11.102
rot	CP000255.1_1890756-1891256	501	10.1128/microbiolspec.GPP3-0031-2018.
rsbU	CP000255.1_2186667-2187668	1002	10.1128/microbiolspec.GPP3-0031-2018.
rsbV	CP000255.1_2186222-2186548	327	10.1128/microbiolspec.GPP3-0031-2018.
rsbW	CP000255.1_2185741-2186220	480	10.1128/microbiolspec.GPP3-0031-2018.
saeP	CP000255.1_768717-769157	441	10.1128/microbiolspec.GPP3-0031-2018.
saeQ	CP000255.1_767902-768375	474	10.1128/microbiolspec.GPP3-0031-2018.
saeR	CP000255.1_767241-767927	687	10.1128/microbiolspec.GPP3-0031-2018.
saeS	CP000255.1_766186-767241	1056	10.1128/microbiolspec.GPP3-0031-2018.
sak	CAA24957.1	492	10.1016/j.biocel.2005.07.005
sasA	CP000255.1_2811367-2818182	6816	10.1038/nrmicro3161
sasB	CP023390.1_2282772-2290208	7437	10.1038/nrmicro3161
sasC	CP000255.1_1877908-1884468	6561	10.1038/nrmicro3161
sasD	CP000255.1_155169-155894	726	10.1038/nrmicro3161
sasE	CP000255.1_1124491-1125543	1053	10.1038/nrmicro3161
sasF	CP000255.1_2798561-2800468	1908	10.1038/nrmicro3161
sasG	AP017320.1_2614378-2617725	3348	10.1038/nrmicro3161
sasH	CP000255.1_31026-33344	2319	10.1038/nrmicro3161
sasI	CP000255.1_1844216-1846903	2688	10.1038/nrmicro3161
sasJ	CP000255.1_1122351-1124288	1938	10.1038/nrmicro3161
sasK	CP001844.2_2683217-2683852	636	10.1038/nrmicro3161
sasX	AXM43800.1	606	10.1038/nrmicro3161
sbi	NC_007795.1_2487383-2488693	1311	10.1038/nrmicro3161
scn	BAB58104.1	351	10.1186/s12864-017-3516-x

scn-b	NZ_CP027476.1_1153847-1154197	351	NA
scn(eq)	LT671578.1_c3610-3266	345	10.1074/jbc.RA117.000599
sdrC	AP009351_601668-604511	2844	10.1128/JCM.44.3.1135–1138.2006
sdrD	AP009351_604878-608825	3948	10.1128/JCM.44.3.1135–1138.2006
sdrE	AP009351_609219-612719	3501	10.1128/JCM.44.3.1135–1138.2006
sea	NZ_WHJU01000002.1_73248-74021	774	10.3390/toxins2071751
seb	AB462486.1	801	10.3390/toxins2071751
sec	AF217235.1	816	10.3390/toxins2071751
secG	BAB56940.1	234	10.1128/JB.01452-09
sed	NZ_SHEG01000015.1_6319-7095	777	10.3390/toxins2071751
sedv3	KX168621.1	540	10.3390/toxins8060169
see	M21319.1	774	10.3390/toxins2071751
seg	AY920259.1	777	10.3390/toxins2071751
seh	U11702.1	726	10.3390/toxins2071751
sei	AF064774.1	729	10.3390/toxins2071751
sej	AB330135.1	807	10.3390/toxins2071751
sek	NZ_WKHU01000012.1	729	10.3390/toxins2071751
sel	NZ_RQIP01000002.1	723	10.3390/toxins2071751
sem	NZ_VMNG01000012.1	720	10.3390/toxins2071751
sen	NZ_VMNG01000012.1	756	10.3390/toxins2071751
seo	AF285760.1_370-1155	786	10.3390/toxins2071751
sep	NC_026016.1_5225_6085	861	10.3390/toxins2071751
seq	NZ_WKHU01000012.1	729	10.3390/toxins2071751
ser	AB330135.1	780	10.3390/toxins2071751
ses	AB330135.1	774	10.3390/toxins2071751
set	AB330135.1	651	10.3390/toxins2071751
seu	AY205306.1	786	10.3390/toxins2071751
sev	EF030427.1	720	10.3390/toxins2071751
sew	NZ_LR134085.1	705	10.3390/toxins2071751
sex	HQ850971.1	612	10.1128/IAI.00505-18
sey	AB924045.1	666	10.1128/IAI.00505-18
sez	NZ_WKIO01000001.1_161741_162520	780	10.1128/IAI.00505-18
shetA	AB036768.1_169-1089	921	10.3390/toxins2051148
shetB	AB036767.1_193-999	807	10.3390/toxins2051148
sigA	CP000255.1_1671296-1672402	1107	10.1046/j.1365-2443.2003.00668.x
sigB	CP000255.1_2184996-2185766	771	10.1046/j.1365-2443.2003.00668.x
sigH	CP000255.1_580244-580813	570	10.1046/j.1365-2443.2003.00668.x
sigS	AAW38354.1	471	10.1371/journal.pone.0003844
smpB	BAB56944.1	465	10.1016/j.febslet.2010.09.024
sodA	NC_002745.2_1588022_1588621	600	10.1128/microbiolspec.GPP3-0031-2018.
spa	CP000255.1_128167-129693	1527	10.1371/journal.ppat.1006917
spdC	NC_007795.1_2401431-2402690	1260	10.1128/IAI.69.3.1521–1527.2001
splA	AF271715.1_1001-1708	708	10.1128/IAI.69.3.1521–1527.2001
splB	AF271715.1_1833-2555	723	10.1128/IAI.69.3.1521–1527.2001
splC	AF271715.1_2613-3332	720	10.1128/IAI.69.3.1521–1527.2001
splD	AF271715.1_3453-4172	720	10.1128/IAI.69.3.1521–1527.2001
splE	AF271715.1_4330-5046	717	10.1128/IAI.69.3.1521–1527.2001
splF	AF271715.1_5197-5916	720	10.1371/journal.ppat.1000927
sprD	NC_002745.2_2007031-2007178	148	10.1128/microbiolspec.GPP3-0031-2018.
srrA	CP000255.1_1596906-1597631	726	10.1128/microbiolspec.GPP3-0031-2018.
srrB	CP000255.1_1595174-1596940	1767	10.1128/microbiolspec.GPP3-0031-2018
sspA	CAA68434.1	1011	10.1128/microbiolspec.GPP3-0031-2018
sspB	BAB57209.1	1182	10.1128/microbiolspec.GPP3-0031-2018
sspC	AAG45845.1	330	doi.org/10.1016/j.fm.2018.01.007
sspP	CAD61962.1	1167	doi.org/10.1016/j.fm.2018.01.007
tarM	BA000033.2_1011181-1012662	1482	10.1016/j.ijmm.2013.10.009
tarS	BA000033.2_280303-282024	1722	10.1016/j.ijmm.2013.10.009
tpiA	CAG39839.1	762	10.1186/s12864-017-3516-x
tst	NZ_BLIF01000001.1	705	10.1128/JB.00146-10
vwb	AY032850.1	1527	10.1099/00221287-148-7-2037
xerC	CP000255.1_1256719-1257615	897	10.1128 /IAI.01462-15.
xerD	NC_007795.1_1516996-1517883	888	10.1128 /IAI.01462-15.
yjbH	CP000255.1_991877-992683	807	10.1128/ IAI.00155-19.
yjbI	CP000255.1_992706-993071	366	10.1128/ IAI.00155-19.

NA: not avaiable

Table S4 List of the reported SCC*mec* elements

SCC*mec* type	Accession number	Reference (doi)
SCC*mec* type I	AB033763.2	10.1128/AAC.45.5.1323-1336.2001
SCC*mec* type IIa	D86934.2	10.1128/AAC.45.5.1323-1336.2001
SCC*mec* type IIb	AB127982.1	10.1128/JCM.43.7.3364-3372.2005
SCC*mec* type IIE	AJ810120.1	10.1128/AAC.49.5.2070-2083.2005
SCC*mec* type III	AB037671.1	10.1128/AAC.45.5.1323-1336.2001
SCC*mec* type IVa	AB063172.2	10.1128/aac.46.4.1147-1152.2002
SCC*mec* type IVb	AB063173.1	10.1128/aac.46.4.1147-1152.2002
SCC*mec* type IVc	AB096217.1	10.1128/aac.46.4.1147-1152.2002
SCC*mec* type IVE	AJ810121.1	10.1128/AAC.49.5.2070-2083.2005
SCC*mec* type IVg	DQ106887.1	10.1093/jac/dki306
SCC*mec* type IVi	AB425823.1	10.1093/jac/dkn435
SCC*mec* type IVj	AB425824.1	10.1093/jac/dkn435
SCC*mec* type V	AB121219.1	10.1128/AAC.48.7.2637-2651.2004
SCC*mec* type V-VT	AB512767.1	10.1007/s10156-011-0223-4
SCC*mec* type VI	AF411935.3	10.1128/AAC.00629-06
SCC*mec* type VII	AB373032.1	10.1128/AAC.00087-08
SCC*mec* type VIII	FJ670542.1	10.1128/JCM.00766-09
SCC*mec* type X	AB505630.1	10.1128/AAC.01475-10
SCC*mec* type IX	AB505628.1	10.1128/AAC.01475-10
SCC*mec* type XI	FR821779.1	10.1016/S1473-3099(11)70126-8
SCC*mec* type XII	KR187111.1	10.1128/AAC.01692-15
SCC*mec* type XIII	MG674089.1	10.1016/j.meegid.2018.03.013
SCC*mec* type XIV	LC440647.1	10.1093/jac/dkz406

Table S5 The Fisher exact test of the distribution of ARGs and VFs in pig-farm and hospital MRSA populations

ID	Positive pigfarm isolates (N = 285)	Positive hospital isolates (N = 198)	*p* -value
**ARGs**			
aac6-Aph2	280	57	2.87E-67
aadD	259	1	4.61E-105
ant6-Ia	5	83	1.50E-31
apH-Stph	285	198	1
aph(3'')-III	4	84	3.64E-33
sat4A	0	17	1.71E-07
spc	0	57	3.37E-25
spw	274	0	1.83E-123
blaI	283	173	1.14E-08
blaR1_Bacilli	282	172	2.87E-08
blaZ	282	173	6.79E-08
mecA	285	198	1
mecI	0	57	3.37E-25
far1	0	7	0.001825661
FosB	277	77	7.57E-50
norA	285	198	1
cfr(A)	1	0	1
erm(A)	0	29	1.60E-12
erm(B)	7	71	8.33E-24
erm(C)	274	52	5.95E-64
lin(A)	4	0	0.147912002
lnu(B)	274	0	1.83E-123
msr(A)	0	1	0.409937888
cat-pC221	0	2	0.167547228
cat-pC223	0	10	0.00011684
dha1	285	198	1
fexA	273	0	3.19E-122
tet(38)	285	198	1
tetK	3	33	5.72E-11
tetL	279	0	2.66E-130
tetM	0	56	1.01E-24
dfrG	277	5	2.36E-117
**VFs**			
FLIPr-like	5	92	2.94E-36
FLIPr	3	77	9.25E-31
aaa	285	198	1
arlR	285	198	1
arlS	285	198	1
atl	285	198	1
bacA	285	198	1
bbp	0	5	0.011233312
bsaA1	1	54	3.34E-22
bsaA2	1	54	3.34E-22
cggr	285	198	1
chp	2	122	3.42E-58
clfA	283	180	5.06E-06
clfB	271	102	3.92E-30
clpB	285	198	1
clpC	285	198	1
clpL	281	97	2.01E-42
clpP	285	198	1
clpQ	285	198	1
clpX	285	198	1
clpY	285	198	1
cna	2	37	1.65E-13
cna_1	1	51	8.07E-21
coa_type2	0	20	9.92E-09
coa_type3	0	11	4.64E-05

coa_type4	0	43	1.11E-18
coa_type5	0	4	0.027735859
coa_type7	5	88	3.91E-34
crtM	285	198	1
crtN	285	198	1
crtO	285	198	1
dltA	285	198	1
dltB	285	198	1
dltC	285	198	1
dltD	285	198	1
eap	282	151	9.37E-17
ebh	282	173	6.79E-08
ebpS	285	189	0.000293026
edin-C	0	5	0.011233312
efb	282	173	6.79E-08
emp	282	173	6.79E-08
eno	285	198	1
est	285	198	1
eta	0	2	0.167547228
etb	0	5	0.011233312
etc	285	198	1
fnbpA	0	52	7.92E-23
fnbpB	270	23	1.66E-85
gapA1	285	198	1
gpmI	285	198	1
hlb	285	198	1
hld	285	198	1
hlgA	285	198	1
hlgB	285	198	1
hlgC	285	198	1
hly	285	197	0.409937888
hysA	285	170	4.29E-12
icaA	285	198	1
icaB	285	198	1
icaC	284	198	1
icaD	285	198	1
icaR	285	198	1
isdC	285	198	1
katA	285	198	1
lexA	285	198	1
lip1	285	198	1
lip2	285	198	1
lukA(G)	284	176	2.13E-08
lukB(H)	284	176	2.13E-08
lukD	1	79	1.61E-34
lukE	1	79	1.61E-34
lukF	2	34	2.89E-12
lukS	2	34	2.89E-12
mgrA	285	198	1
mprF	285	198	1
nuc	285	198	1
oatA	285	198	1
pgk	285	198	1
psm-beta1	285	198	1
psm-beta2	285	197	0.409937888
psm-mec	0	57	3.37E-25
psmA1	285	198	1
psmA2	285	198	1
psmA3	285	198	1
psmA4	285	198	1
rnr	285	198	1
rot	285	198	1
rsbU	285	198	1
rsbV	285	198	1
rsbW	285	198	1
saeP	285	198	1
saeQ	285	198	1
saeR	285	198	1
saeS	285	198	1
sak	4	150	9.49E-76
sarA	285	198	1
sarR	285	198	1

sarS	285	198	1
sarT	5	168	1.70E-90
sarU	5	167	1.66E-89
sarZ	285	198	1
sasA	8	173	1.63E-91
sasB	268	196	0.007143115
sasC	284	169	3.08E-11
sasD	281	109	8.55E-36
sasE	285	196	0.167547228
sasF	284	198	1
sasG	0	61	3.94E-27
sasH	284	197	1
sasI	285	193	0.011233312
sasJ	285	198	1
sasK	278	45	3.70E-74
sasX	0	3	0.068272883
sbi	285	198	1
scn-b	278	88	3.34E-44
scn	5	162	9.66E-85
sdrC	278	146	1.56E-15
sdrD	280	176	2.04E-05
sdrE	284	162	3.51E-14
sea	3	48	3.47E-17
seb	4	68	3.14E-25
sec	0	42	3.13E-18
secG	285	198	1
sed	0	1	0.409937888
seg	114	51	0.00127275
seh	1	11	0.000354888
sei	258	51	8.22E-51
sej	0	1	0.409937888
sek	5	88	3.91E-34
sel	0	42	3.13E-18
sem	258	52	4.10E-50
sen	117	51	0.00065209
seo	277	52	2.59E-67
sep	0	4	0.027735859
seq	5	88	3.91E-34
ser	0	1	0.409937888
seu	250	51	2.29E-45
sew	285	198	1
sex	282	192	0.169846569
sey	281	81	4.68E-52
sez	0	5	0.011233312
sigA	285	198	1
sigB	285	198	1
sigH	285	198	1
sigS	282	165	5.72E-11
smpB	285	198	1
sodA	285	198	1
spa	275	143	1.22E-14
spdC	285	198	1
splA	1	79	1.61E-34
splB	1	79	1.61E-34
splC	1	79	1.61E-34
splD	1	44	1.15E-17
splE	1	25	1.33E-09
splF	0	80	8.36E-37
sprD	5	164	1.30E-86
srrA	285	198	1
srrB	285	198	1
ssl10	282	165	5.72E-11
ssl11	0	52	7.92E-23
ssl12	282	165	5.72E-11
ssl13	282	165	5.72E-11
ssl14	282	172	2.87E-08
ssl1	7	139	3.09E-63
ssl2	282	165	5.72E-11
ssl3	282	161	1.43E-12
ssl4	262	157	0.000105051
ssl5	282	167	3.49E-10
ssl6	277	52	2.59E-67

ssl7	282	165	5.72E-11
ssl8	282	165	5.72E-11
ssl9	285	191	0.001825661
sspA	285	198	1
sspB	285	198	1
sspC	285	198	1
sspP	285	197	0.409937888
tarM	1	68	6.42E-29
tarS	285	190	0.000732566
tpiA	285	198	1
tst	0	28	4.29E-12
vwb	1	64	5.91E-27
xerC	285	198	1
xerD	285	198	1
yjbH	285	198	1
yjbI	285	198	1

PFA-ARGs and HA-VFs were highlighted with yellow color.
